# Assessing CO_2_ Adsorption on Amino-Functionalized Mesocellular Foams Synthesized at Different Aging Temperatures

**DOI:** 10.3389/fchem.2020.591766

**Published:** 2020-11-16

**Authors:** Enrique Vilarrasa-García, Juan Antonio Cecilia, Pedro Augusto S. Moura, Diana C. S. Azevedo, Enrique Rodríguez-Castellón

**Affiliations:** ^1^GPSA-Grupo de Pesquisa em Separações por Adsorção, Departamento de Engenharia Química, Universidade Federal Do Ceará, Campus Do Pici, Fortaleza, Brazil; ^2^Departamento de Química Inorgánica, Cristalografía y Mineralogía, Facultad de Ciencias, Universidad de Málaga, Málaga, Spain

**Keywords:** silica, mesocellular foam, synthesis, amino groups, CO_2_ adsorption

## Abstract

A wide variety of solid sorbents has recently been synthesized for application in CO_2_ adsorption. Among them, mesoporous silicas deserve attention because of their ability to accommodate large concentrations of different chemicals as a consequence of their surface chemistry and tunable pore structure. Functionalized materials exhibit promising features for CO_2_ adsorption at high temperatures and low CO_2_ concentrations. This work aimed to assess the influence of the textural properties on the performance of CO_2_ adsorption on functionalized mesoporous silica. With this goal, several mesoporous silica foams were synthesized by varying the aging temperature, obtaining materials with larger pore diameter. Thus, the synthesized materials were functionalized by grafting or impregnation with 3-aminopropyltriethoxysilane, polyethylenimine, and tetraethylenepentamine as amine sources. Finally, the amino functionalized materials were assessed for CO_2_ capture by means of equilibrium adsorption isotherms at 25, 45, and 65°C. Among the most outstanding results, high aging temperatures favor the performance of impregnated materials by exposing greater pore diameters. Low or intermediate temperatures favor grafting by preserving an appropriate density of silanol groups.

## Introduction

In the last centuries, the increase in world population along with the industrial developments have caused an exponential increase in energy consumption, mainly from the use of fossil fuels as oil and coal. The combustion of these fossil fuels has led to an increase in polluting emissions, which generates serious consequences to the Earth, such as the rupture of the ozone layer, the formation of acid rain and/or the intensification of the Greenhouse Effect (GE). In the case of the GE, CO_2_ emissions, which has increased tremendously since the beginning of the industrial revolution, have caused a progressive global warming (Serreze, 2010; Intergovernmental Panel on Climate Change. Working Group III Edenhofer, 2014). As a consequence of the rising temperature, strong climate changes, such as the glaciers melting and/or the increase in periods of droughts and floods, are affecting agriculture and stockbreeding, causing economic losses and a greater social imbalance (Intergovernmental Panel on Climate Change. Working Group III Edenhofer, 2014). Considering this global problem, some governments propose stringent environmental regulations to minimize anthropogenic CO_2_ emissions, and thus lessen the consequences of GE (Intergovernmental Panel on Climate Change. Working Group III Edenhofer, 2014).

The quest for the mitigation of global warming has led to the development of different strategies aimed at decreasing the emissions of anthropogenic CO_2_. Nowadays, the Carbon Capture and Storage (CCS) processes are the most sustainable technology to mitigate CO_2_ emissions (Pera-Titus, 2014). The most expensive step in CCS is the CO_2_ capture, accounting for 50–90% of the total cost (Pera-Titus, 2014). The most commonly used sorbents in post-combustion CO_2_ capture are based on liquid amine absorption, such as monoethanolamine (MEA), diethanolamine (DEA), and/or methyldiethanolamine (MDEA) as sorbent. Although these amines lead to high efficiency in CO_2_ capture, there are serious disadvantages related to the high levels of equipment corrosion and the high energy consumption required in the regeneration step of the amine, raising the total cost of the method (Oyenekan and Rochelle, 2007; Choi et al., 2009). Several processes have been proposed to replace traditional amine absorption technologies, such as membrane selective permeation or cryogenic distillation; however, the high energy penalty in the case of cryogenic distillation and poor driving force for separation under low CO_2_ concentration limits their application for large-scale (Oyenekan and Rochelle, 2007; Choi et al., 2009; Samanta et al., 2012). Another approach to CO_2_ capture is the use of porous materials with such a small pore size that can act as a molecular sieve through solid-gas interactions (Ma'mun et al., 2007). Previous investigations have reported high CO_2_ adsorption capacity for microporous materials, such as activated carbons (Guo et al., 2006; Wickramaratne and Jaroniec, 2013; Moura et al., 2018), zeolites (Siriwardane et al., 2005; Bezerra et al., 2014) or metal organic frameworks (MOFs) (Sumida et al., 2012; Chen et al., 2016). Metal oxides, such as MgO (Hiremath et al., 2017; Hu et al., 2017) and, mainly CaO (Luo et al., 2015; Ridha et al., 2015) or hydrotalcites, which require a prior activation at high temperatures, strongly bind CO_2_ to form carbonates, but high temperatures are required to desorb CO_2_ and restore the oxide species (Hanif et al., 2014; Silva et al., 2017).

Among all adsorbent materials, the use of SiO_2_-based mesoporous materials has emerged as an alternative in CO_2_ capture processes (Pera-Titus, 2014). Despite the lower CO_2_ adsorption capacity in mesoporous materials than those reached in microporous materials, these mesoporous materials can be used as a support to be functionalized, mainly with amino species, to improve CO_2_ capture due to the coexistence of physical interactions and chemical reactions between the functionalized species and CO_2_ molecules (Gargiulo et al., 2014; Wang et al., 2014). Nonetheless, these interactions are weaker than those shown in the case of metal oxides (MgO or CaO), therefore regeneration of the adsorbent is easier (Pera-Titus, 2014). The functionalization with amino groups comes mainly from two different strategies, grafting (or covalent bonding) and/or (physical) impregnation. Grafting takes place by the condensation reaction between aminoalkylsilanes with the silanol groups on the silica surface leading to porous materials with a large number of available -NH_2_ sites to interact with the CO_2_ molecules. Furthermore, these materials can also act as sieves, so the coexistence of chemical and physical interactions in these sorbents can be considered, as reported in previous studies (Sayari et al., 2011; Vilarrasa-García et al., 2014, 2015b; Cecilia et al., 2016). Impregnation is another methodology used in CO_2_ capture processes. This process consists in hosting amine polymers with a large amount of amino groups, such as polyethylenimine (PEI) or tetraethylenepentamine (TEPA), which are stabilized by physical interactions and hydrogen bonds with the silanol and siloxane groups of the porous silica (Pera-Titus, 2014). Since *Mobil*® scientists discovered a new family of porous materials with a regular array of uniform mesopores, denoted as M41S (Beck et al., 1992), the synthesis of mesoporous silica has evolved in the last 25–30 years. The main structures of this family are MCM-41 (hexagonal *P6mm* symmetry), MCM-48 (cubic *Ia3d* symmetry) and MCM-50 (lamellar structure). On the other hand, in 1998, scientists from the University of California Santa Barbara synthesized a group of materials denoted as SBA-*x*. Among them, SBA-15 also displays hexagonal *P6mm* symmetry like MCM-41, but the mesochannels are interconnected between them by micropores. There is greater flexibility in modulating pore volume of SBA-15 and it has thicker walls as compared to MCM-41, resulting in porous materials with superior hydrothermal and mechanical resistance. Thus, SBA-15 has been widely studied as catalytic support to disperse the active phase (Wang et al., 2005; Singh et al., 2018; Ballesteros-Plata et al., 2019). In addition, the silanol groups of the silica surface are prone to be functionalized, increasing the range of applications. On the other hand, the high microporosity attributed to pores connecting the mesochannels also favors their use in gas adsorption.

The textural properties of the SBA-15 can be modulated tailored according to its final application. In order to increase the pore volume of the SBA-15, several swelling agents, such as alkylbenzenes (Feng et al., 2013; Vilarrasa-García et al., 2014, 2015b), alkanes (Kruk and Cao, 2007; Vilarrasa-García et al., 2014), or amines (Sayari et al., 1998), have been used, which enlarge the volume of the surfactant micelle used as template. Larger pores are expected to cause a decrease in diffusion resistance along the mesochannels. On the other hand, the incorporation of fluoride species can also limit the aggregation of silica in the synthetic step, reducing the length of the mesochannels and frequently leading to mesocellular foams (MCFs). Although MCFs lose the classical hexagonal long-range pore ordering, reducing mesochannels length can contribute to decrease diffusional resistances and increases the availability of silanol groups for functionalization (Lettow et al., 2000; Vilarrasa-García et al., 2014, 2015b; Cecilia et al., 2016). In the present work, several MCFs with different pore sizes and pore volumes have been synthesized to assess a correlation between the textural properties of these MCFs with their CO_2_ adsorption capacity. To improve the adsorption capacity, these porous silicas have been functionalized by grafting with 3-aminopropyltriethoxysilane (APTES) and by impregnation with TEPA and PEI. In addition, this work analyzes the presence of two CO_2_ adsorption sites attributed to physical and chemical interactions.

## Experimental

### Materials

The reagents employed in the synthesis of the MCFs were hydrochloric acid (HCl) (VWR, 37%), triblock copolymer, Pluronic P123, (PEO_20_PPO_70_PEO_20_) with an average molecular weight of 5,800 g mol^−1^ (*Aldrich*^®^), as directing agent structure; tetraethylorthosilicate (TEOS) (*Aldrich*®, 98%), as silicon source; 1,3,5-trimethylbenzene (TMB) (*Aldrich*^®^, 98%), as pore expander and ammonium fluoride (NH_4_F) (*Aldrich*^®^) to limit the growth of the silica mesochannels.

The functionalization of the MCFs with amine groups was carried out using 3-aminopropyltriethoxysilane (APTES) (*Aldrich*^®^, 98%) and toluene (*Aldrich*^®^, 99.5%) in the case of the grafting. The impregnation protocol was performed using branched polyethylenimine (PEI) (average Mn ≈ 600, *Aldrich*^®^) and tetraethylenepentamine (TEPA) (*AcrosOrganics*^®^, 98%) dissolved in methanol (*Aldrich*^®^, 99.9%).

The gases used in the textural characterization and adsorption experiments were He (*AirLiquide*^®^, 99.999%,), N_2_ (*AirLiquide*^®^, 99.9999%), and CO_2_ (*AirLiquide*^®^, 99.998%).

### Synthesis of the Porous Silicas (MCFs)

The synthesis of the MCFs was carried out following the procedure described by Santos et al. with slight modifications (Santos et al., 2016). Briefly, the template (P123) and NH_4_F were dissolved in an acid solution of HCl 1.7 molL^−1^ by stirring at 40°C. After total dissolution of the template, TMB, used as pore expanding agent, was added to the mother solution dropwise. After 30 min, TEOS, the silicon source, was also added to the mother liquor. The final gel has the following molar ratio: 1 P123: 55 SiO_2_: 48 TMB: 350 HCl: 1.8 NH_4_F: 11100 H_2_O. The obtained gel was stirred at 40°C for 24 h and was then transferred to a Teflon-lined autoclave, where the gel was aged for 72 h from room temperature (rt) to 120°C. After that, the gel was filtered and washed with distilled water and dried overnight at 80°C. Finally, the solid was calcined at 550°C to remove the template with a heating rate of 1°C min^−1^, maintaining the calcination temperature for 6 h.

### Functionalization of the MCFs With Amine Groups

The incorporation of amine groups by grafting was carried out following the procedure described by Hiyoshi et al. (2004, 2005). In a typical grafting process, 0.3 g MCF were dried at 110°C under He flow. Then, the dried adsorbent was placed in a three-neck flask with a solution of 20% vol. APTES in toluene (15 mL) and was treated at 110°C for 24 h under reflux. Finally, the functionalized solid was filtered, washed with toluene and dried under air flow at 110°C.

The functionalization by impregnation was carried out following the methodology proposed elsewhere (Sanz et al., 2010; Vilarrasa-García et al., 2017a). Briefly, for each impregnation, 0.3 g MCF were dried at 110°C overnight and then, the dried solid was added to a solution of methanol/amine-rich polymer (PEI or TEPA). The mass ratio methanol/porous silica was maintained constant at 8:1 for each sample, while the methanol/amine-rich polymer was 10:1, leading to silica with an impregnation of 50 wt.% in all cases.

The samples were labeled as MCF-*x*-*yz*, where *x* is the aging temperature in the synthesis step, *y* is the amount of functionalized species: vol.(%) in the case of the APTES and the wt.(%) for loaded PEI or TEPA and *z* represents the amine source (i.e., A for APTES, P for PEI, and T for TEPA. The codes for samples labeling are summarized in Table 1.

**Table 1:** Summary of all synthetized samples.

**Sample code**	**Description**		
	**Synthesis Temp. (°C)**	**Functionalization (Group/Technique)**	**Functionalization amount**
MCF-RT, MCF-60, MCF-80, MCF-100, MCF-120, MCF-140	room temp., (60–140)	-	-
MCF-RT 20A, MCF-60 20A, MCF-80 20A, MCF-100 20A, MCF-120 20A, MCF-140 20A	room temp., (60–140)	APTES/grafting	20.0 vol.%
MCF-RT 50P, MCF-60 50P, MCF-80 50P, MCF-100 50P, MCF-120 50P, MCF-140 50P	room temp., (60–140)	PEI/impregnation	50.0 wt.%
MCF-RT 50T, MCF-60 50T, MCF-80 50T, MCF-100 50T, MCF-120 50T, MCF-140 50T	room temp., (60–140)	TEPA/impregnation	50.0 wt.%

### Characterization Techniques

Small-angle X-ray Scattering (SAXS) measurements were performed in a D8 DISCOVER-Bruker instrument operating at 40 kV and 40 mA. Powder patterns were recorded in capillary-transmission configuration by using a Göbel mirror (CuKα1 radiation) and the LYNX eye detector. The powder patterns were measured between 0.2 and 10° in 2θ with a total measuring time of 120 min. The morphology of the synthesized materials was studied by Transmission Electron Microscopy (TEM), using a FEI Talos F200X equipment.

The textural parameters were evaluated from N_2_ adsorption-desorption isotherms at −196°C, as measured in an automated ASAP 2020 system, by *Micromeritics*^®^. Prior to the measurements, all samples were outgassed overnight at 110°C and 10^−4^ mbar. The specific surface area was determined by the Brunauer-Emmett-Teller equation (BET) using the adsorption data in the range of relative pressures at which conditions of linearity and considerations regarding the method were fulfilled taking into account that the N_2_ cross section is 16.2 Å^2^ (Brunauer et al., 1938). Micropore surface areas were obtained by de Boer's t-plot method (de Boer et al., 1966). The pore size distribution was determined from the desorption branch of the isotherm using the Non-local Density Functional Theory (NLDFT) (Landers et al., 2013). The total pore volume was calculated from adsorbed N_2_ at relative pressure (P/P_0_) = 0.996.

The FTIR spectra were collected in a Varian 3100 Fourier Transform Infrared (FTIR) spectrometer equipped with a Diffuse Reflectance Infrared Fourier Transform (DRIFT) cell. The interferograms consisted of 200 scans, and the spectra were collected using a KBr spectrum as background. For each analysis, about 30 mg of sample was mixed with KBr in a weight ratio of 1:9 and then the spectrum was collected.

The nitrogen content after the functionalization step was determined by elemental chemical analysis using a LECO CHNS 932 analyzer through the combustion of the samples at 1,100°C in pure oxygen.

### CO_2_ Adsorption Measurements

CO_2_ adsorption-desorption isotherms were measured with a Micromeritics ASAP 2020 Analyzer (i.e., volumetrically) between 25 and 65°C. Prior to the measurements, samples were outgassed at 110°C and 10^−4^ mbar until total outgassing was reached.

### Equilibrium Model

The CO_2_ adsorption isotherms on pure MCF were modeled by Langmuir model Equation (1) (Langmuir, 1918; Do, 1998). In the case of CO_2_ adsorption on functionalized MCF, the Dual Site Langmuir Model – DsL Equation (2) was applied (Serna-Guerrero et al., 2010). One of the sites is ascribed to the interaction of CO_2_ molecules with silanol groups and/or hydroxyl groups (physisorption sites – 1, with lower value of b_i_) while the second site is ascribed to the interaction of CO_2_ with amines-species located on the surface or inside mesoporous silica (chemisorption sites – 2, with higher value of b_i_). The Dualsite Langmuir model Equation (1) was used to qualify the interaction between adsorbent and adsorbate. This model is a variation of Langmuir model.

(1) $$\begin{array}{l} q=\frac{q_{m}bP}{1+bP} \end{array}$$

(2) $$\begin{array}{l} q=\frac{q_{m1}b_{1}P}{1+b_{1}P}+\frac{q_{m2}b_{2}P}{1+b_{2}P} \end{array}$$

where q_m_ (mmol g^−1^) represents the maximum adsorbed capacity, b (kPa^−1^) is the affinity constant, P is the pressure (kPa).

K_Henry_ (mmol g^−1^kPa^−1^) is the Henry's law constant, taking into account that Langmuir's equation reduces to Henry's law when pressure tends to zero. Thereby the Henry's law can be written as follows Equation (3):

(3) $$\begin{array}{l} q=K_{H}P=q_{mi}b_{i}P \end{array}$$

To estimate the accuracy of each fit, the Average Relative Error (ARE) Equation (4) function based on the difference of the experimental and estimated amount adsorbed was used:

(4) $$\begin{array}{l} ARE\;\left(\%\right)=\frac{1}{N_{T}}\overset{N_{T}}{\underset{i=1}{\sum }}\frac{\left|q_{i,\text{exp}}-q_{i,est}\right|}{q_{i,\text{exp}}}*100 \end{array}$$

where N_T_ is the number of the data points, and q_i,exp_ and q_i,est_ are the experimental and estimated amounts adsorbed, respectively.

## Characterization of the MCFs

**Figure 1:**
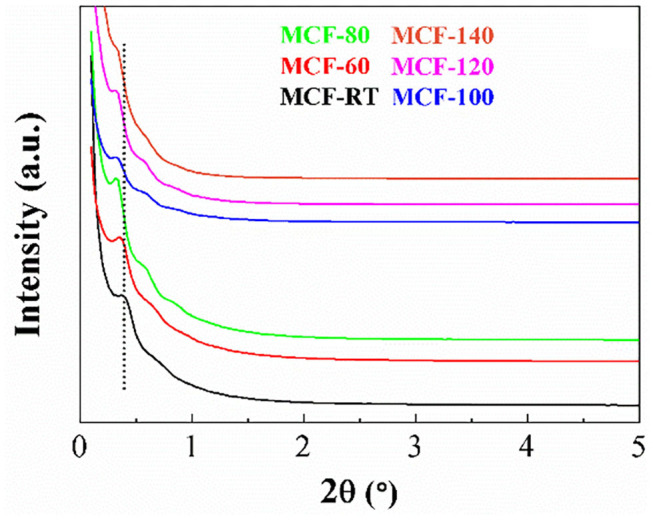
SAXS profiles of the MCFs synthesized with different aging temperature (from room temperature to 140°C).

The arrangement of the MCFs synthesized at different aging temperatures was evaluated by SAXS as shown in Figure 1. In the case of the MCF-RT, it is noteworthy the presence of a band located about 2θ = 0.38°, which is attributed to the (100) reflection and another less intense and broader band located about 2θ = 0.60°, ascribed to the coexistence of the (110) and (200) reflections, which is in concordance with those previously reported in the literature (Lettow et al., 2000; Schmidt-Winkel et al., 2000). An increase of the aging temperature causes a progressive shift of the reflections to lower 2θ values, reaching a value of 0.32° for the (100) reflection in the case of the MCF-140 sample, which indicates the formation of structures with larger pore size. In addition, the MCFs synthesized at intermediates temperatures (60–80°C) display better-defined diffraction profiles, suggesting the formation of structures with higher long-range ordering. Thus, these structures display a third reflection band located about 2θ = 0.84°, which can be attributed to the overlapping of the (210) and (300) reflections (Lettow et al., 2000; Schmidt-Winkel et al., 2000).

The SAXS profile of MCF-100 was chosen as representative sample to compare it with that obtained for SBA-15 obtained under similar synthetic conditions, but without the incorporation of modifying structure agents in the synthetic step (Supplementary Figure 1). It can be observed how the reflections of the SBA-100 are significantly more intense than those shown for MCF-100, which reveals that the SBA-15 presents a more ordered structure than the MCFs. Regarding this evidence, several authors (Nguyen et al., 2008; Santos et al., 2016) have pointed out that the incorporation of a swelling agent, as TMB, causes an increase of the volume occupied by the hydrophobic template in the micelle core. This gives rise to structures with larger pore size, as suggested by the shift of the (100) reflection at lower 2θ values in the case of the MCF-100; however, this also leads to a partial loss of the typical hexagonal arrangement of SBA-15. Likewise, the addition of NH_4_F limits the aggregation and growth of the mesochannels, generating structures with lower long-range order (Lettow et al., 2000). Considering both premises, it seems clear that the incorporation of additives leads to modifications in the textural properties of the porous silica; however, it may also display an adverse effect related to the control of the self-assembly manner (Yao et al., 2004).

**Figure 2:**
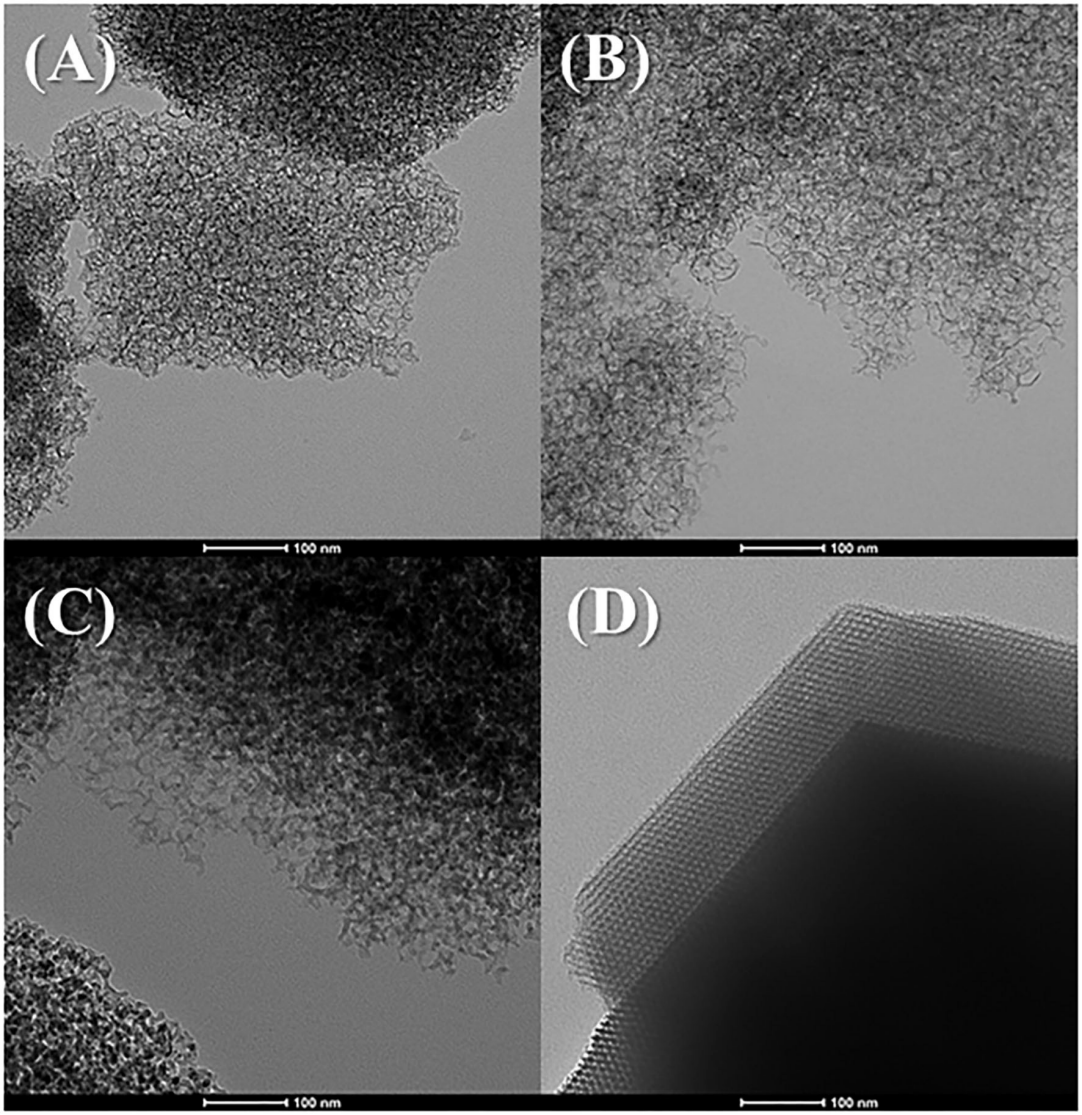
TEM images of the MCFs aged to rt **(A)**, 100°C **(B)**, and 140°C **(C)** and micrography of SBA-15 aged to 100°C **(D)** (scale: 100 nm).

The morphology of the MCFs was evaluated from their transmission electron micrographs (Figure 2). Figures 2A–C reveals the formation of a heterogeneous porous structure with mesocellular foam-like morphology, as was previously described by other authors (Lettow et al., 2000; Vilarrasa-García et al., 2014, 2015b; Cecilia et al., 2016). The increase of the aging temperature in the synthetic step seems to form structures with slightly larger pore size, which is in agreement with the shift at lower 2θ values shown in Figure 1. In the case of MCF-140 (Figure 2C), the obtained structure looks the most disordered, which is also in accordance with Figure 1, since the reflections obtained from SAXS are less intense than for higher aging temperatures. Finally, the comparison between MCF-100 and SBA-100 (Figures 2B,D) confirms that the incorporation of structural modifying agents leads to the formation of wider pores, although it also generates a greater structural disorder, leading to much more heterogeneous structures, as previously suggested from SAXS data in Figure 1.

**Figure 3:**
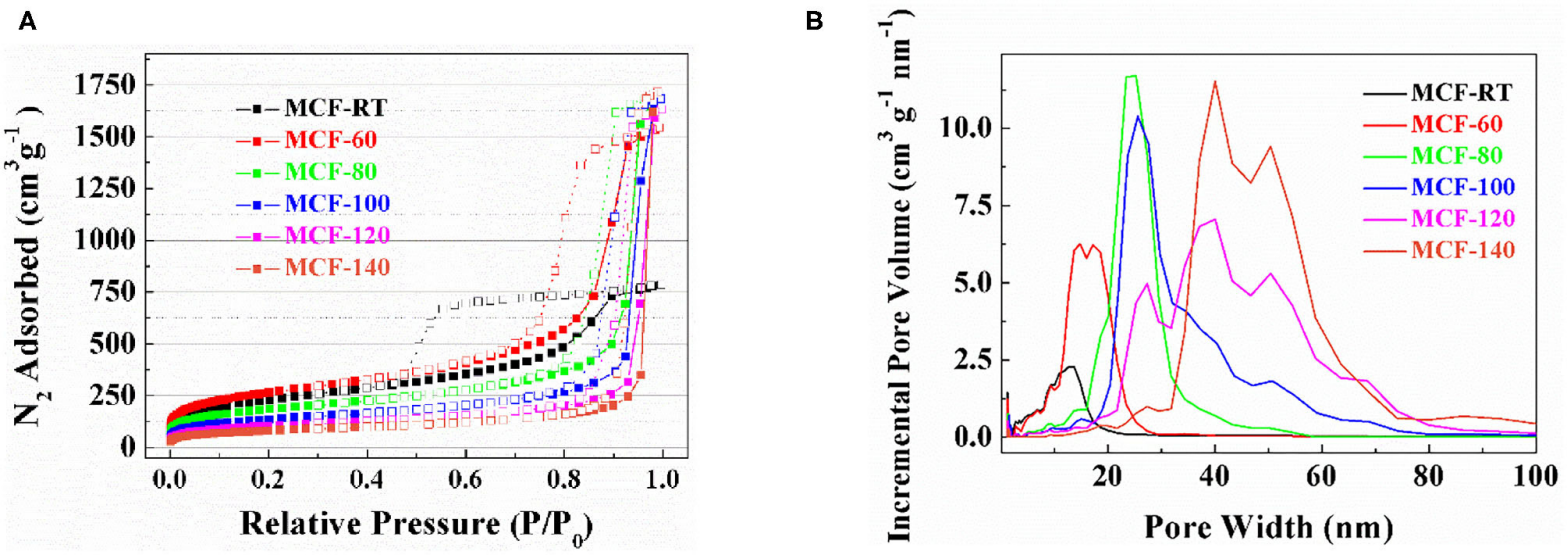
N_2_ adsorption-desorption isotherm at −196°C **(A)** and pore size distribution determined by NLDFT method **(B)** of the MCFs synthesized with different aging temperature (from rt to 140°C).

The textural properties of the MCFs were determined from their N_2_ adsorption-desorption isotherms at −196°C (Figure 3A). According to the IUPAC classification, the isotherm profiles of the samples synthesized with lower aging temperature can be considered as IVa-type, which are ascribed to mesoporous materials (Thommes et al., 2015). An increase of the aging temperature gives rise to the evolution of the isotherm profile to II-type, which is assigned to non-porous or, as in the present case, macroporous materials (Thommes et al., 2015). These data are correlated with their respective hysteresis loops, which are shifted to higher relative pressure as the aging temperature of the MCF increases. The hysteresis loop (Figure 3A) also reveals that MCF-RT displays a hysteresis loop of type H2(a), which is attributed to pore-blocking in a narrow range of pore necks or to cavitation-induced evaporation. The remaining MCFs show hysteresis loops of type H2(b) that are also associated with pore blockage, although the size distribution of neck width is much larger (Thommes et al., 2015).

The specific surface area (S_BET_) was determined by the BET equation (Brunauer et al., 1938). Table 2 shows how the S_BET_ values, as well as the microporosity of the MCFs, decrease according as the aging temperature increases. These data are in agreement with those reported by Galarneau et al. (2001), which pointed out that the use of lower aging temperature favors the interconnection of P-123 micelles in such a way that micropores are generated after calcination of the template (Galarneau et al., 2001). The use of higher aging temperature moves P-123 micelles away from each other so that intercommunication also decreases, leading to a structure with lower microporosity (Galarneau et al., 2001).

**Table 2:** Textural properties of MCF series.

**Sample**	**S_BET_ (m^2^g^−1^)**	**t-plot (m^2^g^−1^)**	**t-plot micropore (cm^3^g^−1^)**	**Pore volume (cm^3^g^−1^)**	**Average pore size (nm)**
MCF-RT	824	217	0.096	1.213	13.2
MCF-60	955	219	0.085	2.386	17.5
MCF-80	661	212	0.075	2.621	19.7
MCF-100	488	158	0.071	2.602	25.2
MCF-120	373	125	0.055	2.522	32.7
MCF-140	293	99	0.044	2.654	42.8

The increase of the aging temperature also causes a shift of the average pore size to higher values. The pore size distribution was more clearly estimated from NLDFT method (Landers et al., 2013) (Figure 3B). The maximum value of the pore size distribution increases with the aging temperature from 13.2 nm for MCF-RT to 41.0 nm for MCF-140. However, the distribution is wide, which indicates the formation of a relatively heterogeneous structure, as was suggested by SAXS and TEM data (Figures 1, 2A–C).

The comparison of N_2_ adsorption-desorption isotherms of SBA-100 and MCF-100 is shown in Supplementary Figure 2. The hysteresis loop of the SBA-100 takes place at lower relative pressure. The isotherm profile of SBA-100 samples can be classified as IVa-type, while the profile of MCF-100 sample is closer to the II-type isotherm, as indicated by the increase of the N_2_-adsorbed at higher relative pressure range. In addition, the presence of long mesochannels in the SBA-100 sample (Figure 2D) leads to a higher S_BET_ value than that shown for the MCF-100 sample. This fact implies the formation of a more ordered structure with narrower pore size distribution in the case of SBA-15 (Supplementary Figure 3), as also suggested by the SAXS data (Figure 1), with a lower pore size in comparison to the MCF-100 sample.

**Figure 4:**
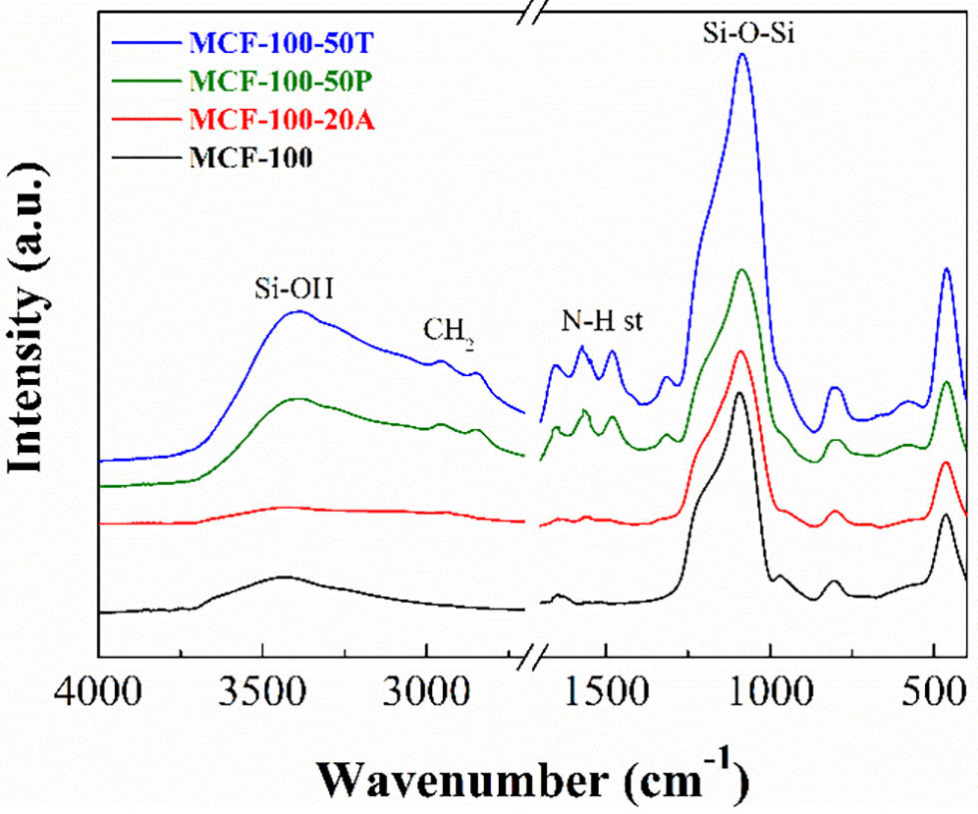
FTIR spectra of the MCF-100, MCF-100 20A, MCF-100 50P, and MCF-100 50T.

As all the adsorbents are silica-based materials and displays similar bands in the FTIR spectra, the MCF-100 sample was chosen to be shown in Figure 4. The main band between 1,300 and 995 cm^−1^ is attributed to the overlap of the Si-O-Si longitudinal asymmetric stretching, located about 1,100 cm^−1^ and Si-O-Si transverse asymmetric stretching, with a maximum about 1,190 cm^−1^ (Borodko et al., 2005). The band centered at 810 cm^−1^ is attributed to Si-O-Si symmetric stretching vibrations and the band located at 470 cm^−1^ is assigned to the Si-O-Si bending vibrations, while the band centered at 970 cm^−1^ is assigned to the Si-O in plane stretching vibrations (Colilla et al., 2010). The FTIR spectrum of MCF-100 also displays a broad band between 3,800 and 2,700 cm^−1^, which is attributed to the water molecules interacting with the silica. In addition, the absence of the bands attributed to silanol groups (3,740 cm^−1^) is noteworthy, probably due to the calcination conditions leading to siloxane species (Burneau et al., 1990).

## CO_2_ Adsorption Data

### CO_2_ Adsorption on MCFs

**Figure 5:**
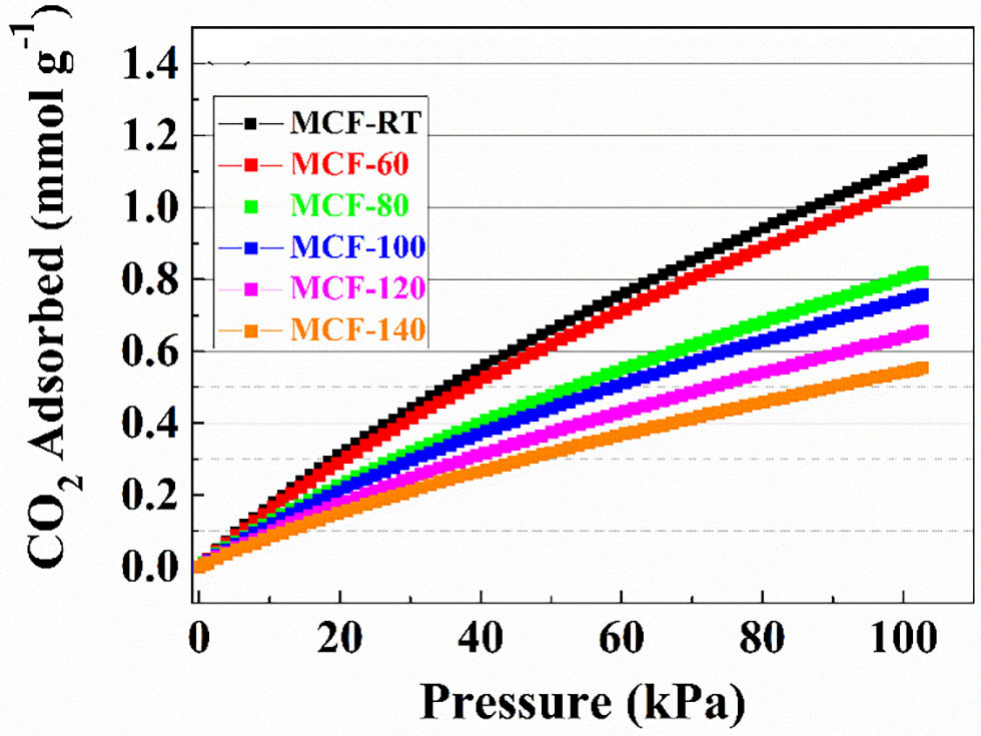
CO_2_ adsorption isotherms at 25°C of the MCFs synthesized with different aging temperature (from rt to 140°C).

The CO_2_ adsorption isotherms obtained for the MCFs aged at different aging temperature are compiled in Figure 5. In all cases, the isotherms show a quasi-linear uptake behavior as the absolute pressure increases. The adsorption data reveal that the adsorbent synthesized at room temperature (MCF-RT) has the highest CO_2_ adsorption capacity, reaching a value of 1.13 mmol g^−1^ at 25°C and 100 kPa. The increase of the aging temperature causes a progressive decrease of the CO_2_ adsorption, the lowest value of 0.55 mmol g^−1^ being obtained for MCF-140 under similar experimental conditions. This trend is attributed to the textural properties of the MCFs since it is well-known that porous materials act as sieve in the CO_2_ selective adsorption (Choi et al., 2009). Thus, those materials with smaller pore sizes and narrower pore size distributions, such as MOFs, zeolites or activated carbons, reach the highest CO_2_ uptakes (Pera-Titus, 2014). The adsorption data shown for the MCFs follow this trend since the porous silicas with higher pore volume into the micropore range, as indicates the *t*-plot values (Table 2) and the pore size distribution (Figure 3B), are more likely to capture CO_2_.

The Langmuir model was applied to fit the experimental data. The adsorption model shows a good fit with the experimental data in the studied pressure range, as indicates the low ARE values. The fitting parameters are summarized in Table 3. These data reveal that the highest q_max_ value is obtained for the MCF-60 samples 2.834 mmol g^−1^. As was indicated previously, the increase of the pore volume also displays a decrease of the q_max_ value, obtaining a lowest value of 1.439 mmol g^−1^ for MCF-140 sample. The b parameter defines the strength of the interaction between the adsorbate (CO_2_) and the adsorbent (porous silica). These data reveal that the sample with the highest CO_2_ adsorption capacity also displays the largest Henry's law constant (K_Henry_) as a consequence of its higher micropore volume (see *t*-plot and micropore volume values in Table 2), which favors a stronger interaction between CO_2_ molecules and the porous structure due to more intense confinement effects. In other words, as the aging temperature increases the K_Henry_ values (Table 3) decrease and this fact could be related to the micropore volume decrease (Table 2).

**Table 3:** Langmuir model fitting parameters for MCF Series Isotherms.

**Sample**	**q_m_ [mmol g^−1^]**	b *10^3^ (kPa^−1^)	**ARE (%)**	${\text{K}}_{\text{Henry}}^{*}$10^2^ (mmolg^−1^kPa^−1^)
MCF-RT	2.792	6.296	1.761	1.758
MCF-60	2.834	5.671	1.478	1.607
MCF-80	2.014	6.329	1.769	1.274
MCF-100	1.891	6.213	1.556	1.175
MCF-120	1.736	5.584	1.963	0.969
MCF-140	1.439	5.785	1.687	0.832

*ARE, average relative error (%)*.

CO_2_ adsorption capacity of the MCF-100 was compared with that obtained for SBA-100 sample (Supplementary Figure 4). These data reveal that SBA-100 displays a CO_2_ adsorption of 0.93 mmol g^−1^ at 25°C and 100 kPa, while MCF-100 only reaches a CO_2_ adsorption of 0.75 mmol g^−1^ at the same conditions. The difference in the CO_2_ uptake may be attributed to their different textural properties. MCF-100 has a heterogeneous porous structure with a higher pore size than SBA-100 (Supplementary Figure 3), the latter consisting of homogeneous and narrower meso channels interconnected between them by micro channels. This explains the higher *t*-plot = 179 m^2^g^−1^ and micropore volume = 0.097 cm^3^g^−1^ of sample SBA-100, which favors its superior CO_2_ adsorption.

### CO_2_ Adsorption on Amino Functionalized MCF

Considering that MCFs are porous silica with wide pore diameter and short length of channels, these materials seem to be appropriate to incorporate a considerable amount of amine-species in such a way that chemical interaction between the adsorbent and CO_2_ can improve the CO_2_ adsorption capacity (Sayari et al., 2011). It is well-known that the most common mechanism for CO_2_ capture in primary or secondary amino functionalized materials involves the formation of a zwitterion (Caplow, 1968; Danckwerts, 1979) through the interaction of CO_2_ with an amine, followed by deprotonation of the zwitterion by a base to produce a carbamate (Donaldson and Nguyen, 1980; Pinto et al., 2011; Mebane et al., 2013; Didas et al., 2014). The typical heats of adsorption reported for this interaction is in the range from 65 to 135 kJmol^−1^ (Potter et al., 2017), depending on the density of amine groups (Alkhabbaz et al., 2014). The mechanism for the interaction between CO_2_ and primary/secondary amine is given below:

Primary amine:

(5) $$\begin{array}{l} \text{First step}:\text{ RN}{\text{H}}_{2}+\text{C}{\text{O}}_{2}↔\text{ RN}{\text{H}}_{2}^{+}\text{CO}{\text{O}}^{-} \end{array}$$

(6) $$\begin{array}{l} \text{Second step}:\text{ RN}{\text{H}}_{2}^{+}\text{CO}{\text{O}}^{-}+\text{RN}{\text{H}}_{2}↔\text{ RNHCO}{\text{O}}^{-}+\text{RN}{\text{H}}_{3}^{+} \end{array}$$

Secondary amine:

(7) $$\begin{array}{l} \text{First step}:\;{\text{R}}_{2}\text{NH}+\text{C}{\text{O}}_{2}↔\;{\text{R}}_{2}\text{N}{\text{H}}^{+}\text{CO}{\text{O}}^{-} \end{array}$$

(8) $$\begin{array}{l} \text{Second step}:\text{ RN}{\text{H}}^{+}\text{CO}{\text{O}}^{-}+{\text{R}}_{2}\text{NH}↔\;{\text{R}}_{2}\text{NCO}{\text{O}}^{-}+{\text{R}}_{2}\text{N}{\text{H}}_{2}^{+} \end{array}$$

Tertiary amines react with CO_2_ producing bicarbonate instead of carbamate (Bishnoi and Rochelle, 2000), with lower heat of adsorption than primary and secondary amines (Ko et al., 2011). The reaction mechanism between CO_2_ and tertiary amine interaction is given below:

(9) $$\begin{array}{l} \text{First step}:\;{\text{R}}_{3}\text{N}+\text{C}{\text{O}}_{2}↔\;{\text{R}}_{3}{\text{N}}^{+}\text{CO}{\text{O}}^{-} \end{array}$$

(10) $$\begin{array}{l} \text{Second step}:\;{\text{R}}_{3}{\text{N}}^{+}\text{CO}{\text{O}}^{-}+{\text{R}}_{3}\text{N}↔\;{\text{R}}_{2}\text{NCO}{\text{O}}^{-}+{\text{R}}_{4}{\text{N}}^{+} \end{array}$$

The efficiency, defined as the ratio of moles of CO_2_ captured to moles of nitrogen in the material, is a useful measure to quantify the effectiveness of adsorbents for CO_2_ capture. In the “zwitterionic” mechanism, under anhydrous conditions, a second amine acts as a base to produce an ammonium carbamate, giving a theoretical maximum efficiency of 0.5. This suggests that getting materials with a high amine density close to each other can improve the capture efficiency.

Thus, the porous silicas were functionalized by grafting with APTES and by impregnation with amine-rich polymers PEI or TEPA. The elemental analysis of functionalized materials is shown in Table 4.

**Table 4:** Elemental analysis (CHN) of amino functionalized materials determined.

**Sample**	**APTES (20% v/v)**		**PEI (50 wt. %)**		**TEPA (50 wt. %)**	
	**%C**	**%N**	**%C**	**%N**	**%C**	**%N**
MCF-RT	6.959	2.681	23.719	14.206	25.291	14.676
MCF-60	9.916	3.335	22.314	13.240	21.094	12.055
MCF-80	6.807	2.593	24.298	14.651	21.353	12.838
MCF-100	7.196	2.718	22.028	13.824	18.681	11.042
MCF-120	7.094	2.667	22.580	13.035	22.636	14.104
MCF-140	6.316	2.396	25.598	14.676	18.441	11.056

#### Functionalization of the MCFs by Grafting With APTES

Once the MCFs were functionalized by grafting with APTES, the incorporation of the nitrogen species was confirmed by CHN and FT-IR.

The elemental analysis (CHN) of the MCFs grafted with APTES shows N-contents between 2.4 and 3.6% (Table 4). These values are in the same range to those reported in other porous silicas described previously (Vilarrasa-García et al., 2015a; Cecilia et al., 2020; Sánchez-Zambrano et al., 2020). As the grafting conditions were the same in all cases, the N-content must be directly related to the amount of available silanol groups of the MCFs since the grafting reaction takes place between the silanol group of the MCFs and APTES in such a way that the MFCs are functionalized with amine groups (Hiyoshi et al., 2004, 2005).

The FT-IR spectrum of the MCF-100 20A displays slight modifications in comparison to the MCF-100 spectrum (Figure 4). Thus, it is noticeable the appearance of two bands located at 1,570 and 1,480 cm^−1^, which are attributed to asymmetric and symmetric bending C-H (Wang et al., 2009). In the same way, the weak bands located about 2,950 and 2,840 cm^−1^ are attributed to asymmetrical and symmetrical C-H stretching vibrations coming from the alkyl-chain of the APTES or the amine-rich polymers (Vilarrasa-García et al., 2015b). The typical symmetrical and asymmetrical N-H stretching, which should appear about 3,370 and 3,270 cm^−1^ (Wang et al., 2009), are not observed probably due to these bands can be masked by the broad band located between 3,700 and 2,600 cm^−1^ that is ascribed to the physisorbed water.

**Figure 6:**
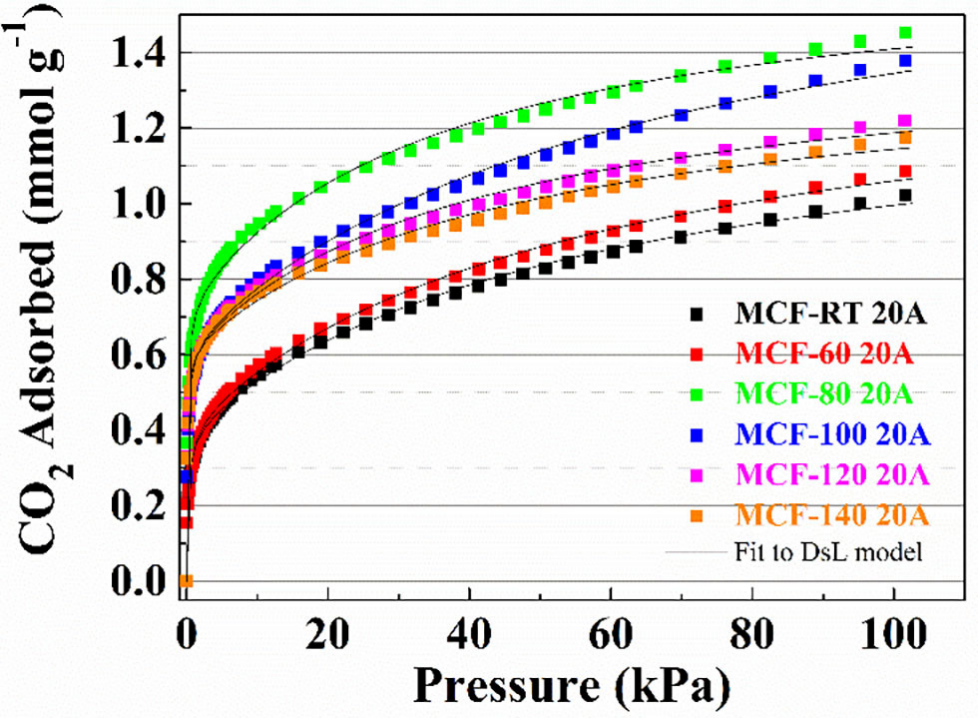
CO_2_ adsorption isotherms at 25°C of the porous silicas (MCFs) synthesized under different aging temperature (from room temperature to 140°C) grafted with APTES. Solid lines for fit to DsL model.

The CO_2_ adsorption capacities of the MCFs functionalized by grafting with APTES are compiled in Figure 6. All isotherms have a more pronounced non-linear character than those observed for MCFs without amine-species. The considerable increase in CO_2_ uptake at low pressure suggests the existence of chemical sites, which adsorb more strongly than physical ones. CO_2_ uptakes in samples functionalized with APTES do not follow the same trends as those observed for the non-functionalized silica samples, in which case the adsorbents with lower pore diameter and pore volume reached higher CO_2_ adsorption capacities (Figure 5). In the case of the MCFs functionalized with APTES, the highest CO_2_ adsorption capacity was obtained for the sample MCF-80 20A, which reaches a value of 1.45 mmol CO_2_ g^−1^ at 100 kPa and 25°C, while adsorbents with narrower pore size, such as MCF-RT, only reaches 1.11 mmol CO_2_ g^−1^. The use of a higher aging temperature favors the increase of the pore size, as indicated in Figure 3B. However, beyond the aging temperature of 80°C, the CO_2_ adsorption capacity decreases, reaching 1.18 mmol g^−1^ for the MCF-140 20A sample.

Adsorption isotherms for the APTES-grafted samples were well fitted to the Dualsite Langmuir model (Table 5), as suggested by the low ARE values. This adsorption model was chosen because it takes into account two adsorption sites ascribed to the physical (Site 1) and the chemical interactions (Site 2). In this adsorption model, the strength of interaction between CO_2_ and the adsorbent, defined by b_i_ values, confirms that the chemical interactions are stronger than physical ones as indicated by the higher b_2_ values as opposed to b_1_values. The grafting of APTES on the MFCs causes a partial blockage of the porous structure although these adsorbents still maintain a remarkable surface area. This fact explains the decreases both the S_BET_ ant *t*-plot values, but these adsorbents still have potential to act as molecular sieves (Table 6). From these data, it is to be expected that those MCFs grafted with APTES, with higher S_BET_ and *t*-plot, should reach the superior CO_2_ uptakes due to higher proportion of physical adsorption sites shown for these materials.

**Table 5:** Dualsite Langmuir model fitting parameters for MCF (with APTES).

**Sample**	**q_m1_ (mmolg^−1^)**	b_1_ *10^3^ (kPa^−1^)	**q_m2_ (mmolg^−1^)**	**b_2_ (kPa^−1^)**	**ARE (%)**
MCF-RT 20A	0.937	18.113	0.395	4.876	2.742
MCF-60 20A	1.022	17.451	0.411	3.834	3.091
MCF-80 20A	0.928	26.543	0.737	8.718	2.537
MCF-100 20A	1.191	25.229	0.627	7.086	2.643
MCF-120 20A	0.819	24.171	0.608	6.734	2.433
MCF-140 20A	0.782	21.654	0.519	5.542	2.401

*ARE, average relative error (%)*.

**Table 6:** Textural properties, nitrogen content, and efficiency of the N-sites for the MCFs synthesized under different aging temperature (from room temperature to 140°C) grafted with APTES.

**Sample**	**S_BET_ (m^2^g^−1^)**	**t-plot (m^2^g^−1^)**	**N (wt.%)**	**q_15_ [mmol g^−1^]**	**q_m2_ [mmol g^−1^]**	**mol CO_2_/mol N (q_m2_)**
MCF-RT 20A	197	20	2.681	0.592	0.395	0.206
MCF-60 20A	225	30	3.335	0.624	0.411	0.173
MCF-80 20A	247	36	3.093	1.000	0.737	0.334
MCF-100 20A	201	27	2.718	0.856	0.627	0.323
MCF-120 20A	140	24	2.667	0.829	0.608	0.319
MCF-140 20A	104	21	2.396	0.621	0.519	0.303

*q_15_, quantity of CO_2_ adsorbed at 15 kPa*.

In previous investigations (Mebane et al., 2013; Hwang et al., 2014; Sánchez-Zambrano et al., 2020), it has been established that the chemical interaction between CO_2_ molecules and the amine-species takes place via a zwitterion intermediate to form carbamate species. These authors reported that the maximum theoretical efficiency of the N-species must reach a CO_2_/N molar ratio of 0.5 in dry conditions (Sanz-Pérez et al., 2016). Taking into account that N-species are only involved in the CO_2_ chemisorption, the efficiency was calculated considering the maximum quantity of CO_2_ adsorbed on chemical sites (q_m2_). The experimental efficiency values of the N-species, shown in Table 6, are between 0.206 and 0.334, and these data are below the theoretical values. In this sense, an efficiency of 0.5 is reported for those adsorbents where all N-species are available on its surface as –NH_2_. However, these –NH_2_ sites can react with other APTES molecules in the grafting step or the APTES molecules grafted can react with neighboring silanol groups forming secondary or ternary amines (Hiyoshi et al., 2005), which leads to a lower efficiency in comparison to the primary amines (Sanz-Pérez et al., 2016).

In order to compare between SBA-15 and MCF synthesized at the same aging temperature (100°C), both adsorbents were functionalized with the same proportion of APTES (Supplementary Figure 5). The obtained data reveal higher CO_2_ adsorption capacity in the case of MCF-100 20A sample, which reaches a value of 1.38 mmol g^−1^, while the SBA-100 20A sample only achieves 0.97 mmol g^−1^. In both cases, the samples adsorb at low absolute pressure in higher proportion than the respective supports, confirming the appearance of chemical adsorption sites. Despite the higher N-content of SBA-100 20A (3.63 wt.%), CO_2_ uptake of this adsorbent is lower. This is also true for the efficiency of the N species since the CO_2_/N molar ratio is 0.25 for SBA-100 20A, considerably below that reached by MCF-100 20A (0.323). These evidences suggest that the accessibility of the N-species is limited for the SBA-100 20A due to the smaller pore size and longer channels as compared to MCFs. As the pore diameter of the channels is narrower, it is also likely that –NH_2_ species interact with neighboring silanol groups to form secondary or ternary amines, which lower capture efficiency as compared to primary amines (Sanz-Pérez et al., 2016).

#### Functionalization of the MCFs by Impregnation With PEI

MCFs were also functionalized by impregnation with amine-rich polymer PEI. The FT-IR spectrum of MCF-100 50P displays the same bands that were previously observed for the MCF-100 20A, although in this case the intensity of these bands is more pronounced (Figure 4). This fact is ascribed to the incorporation of larger amount of C- and N-species by the impregnation method as was shown from the elemental analysis (CHN) (Table 4). The bands located at 2,947 and 2,840 cm^−1^ correspond to the symmetric and non-symmetric stretching vibrations of –CH in PEI. The bands at 1,570 and 1,485 cm^−1^ are related to the symmetric and non-symmetric stretching vibrations of N-H in PEI, indicating that PEI was successfully impregnated on MCF (Chen et al., 2010). These data show a N-content between 13.0 and 14.7 wt.%.

**Figure 7:**
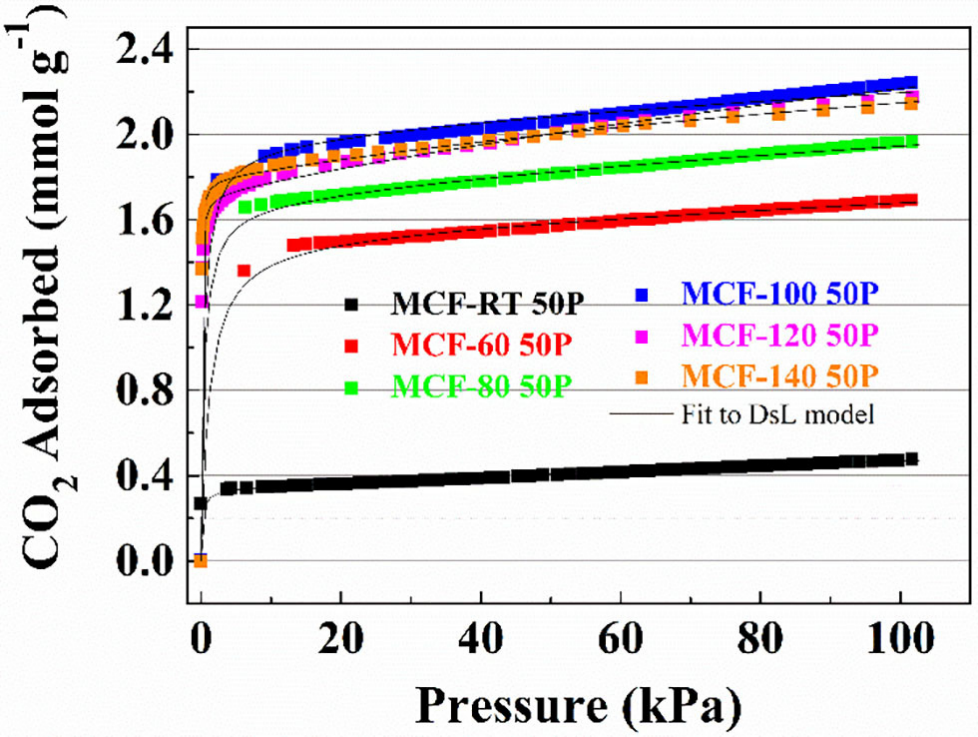
CO_2_ adsorption isotherms at 25°C of the porous silicas (MCFs) synthesized under different aging temperature (from room temperature to 140°C) impregnated with PEI. Solid lines for fit to DsL model.

CO_2_ adsorption data for the MCFs impregnated with PEI are shown in Figure 7. All isotherms exhibit a much more steep profile at low pressures than those for the MFCs and the APTES-grafted MFCs, although this slope slightly varies according to the aging temperature. At low absolute pressures, the adsorption process is governed mainly by chemical interactions while the physical contribution can be considered marginal. The adsorption data (Figure 7) reveals that the CO_2_ uptake increases with rising aging temperatures: from 0.48 mmol CO_2_ g^−1^ for the sample MCF-RT 50P to 2.24 mmol CO_2_ g^−1^ for the sample MCF-100 50P. From this point on, higher aging temperature to increase of pore diameter of the MCF hardly affects the CO_2_ adsorption capacity.

The Dualsite Langmuir model was used to fit the isotherm data (Table 7). The estimated model parameters confirm that adsorbate-adsorbent interaction is much higher in the chemical sites (b_2_) in comparison to the physical sites (b_1_). The textural properties of the samples impregnated with PEI show a drastic decrease in both S_BET_ and *t*-plot values (Table 8), more pronounced than that observed after the grafting process (Table 6). Hence, impregnation of a large amount of amine-rich polymer fills the porous structure. However, as MCFs have a very wide pore diameter, this pore filling is less pronounced than in other porous materials with a smaller pore size (Cecilia et al., 2018, 2020) in which case the amine-rich polymer stacks on the surface of the silica blocking the porous structure and thus decreasing the access to chemisorption sites. This fact can be observed in the data shown in Figure 7 and Table 8: the adsorbents with a lower pore diameter tend to load a similar amount of PEI and CO_2_ adsorption capacity is lower. The efficiency of the N-species from PEI (Table 8) is well below the theoretical values and those reported for the APTES-grafted MCFs. These low efficiency values are possibly due to the poor exposure of stacked amine-rich polymer in the pores as well as the presence of primary, secondary and ternary amines. This is particularly evident for those adsorbents with lower pore diameter.

**Table 7:** Dualsite Langmuir model fitting parameters for MCFs impregnated with PEI.

**Sample**	**q_m1_ (mmolg^−1^)**	b_1_ *10^3^ (kPa^−1^)	**q_m2_ (mmolg^−1^)**	**b_2_ (kPa^−1^)**	**ARE (%)**
MCF-RT 50P	1.055	1.379	0.339	3.876	3.221
MCF-60 50P	1.333	1.483	1.523	0.851	3.088
MCF-80 50P	0.978	3.922	1.676	2.213	5.318
MCF-100 50P	0.911	3.498	1.953	12.262	0.929
MCF-120 50P	1.911	3.532	1.712	19.278	1.676
MCF-140 50P	1.131	4.666	1.788	24.813	1.111

*ARE, average relative error (%)*.

**Table 8:** Textural properties, nitrogen content and efficiency of the N-sites for the MCFs synthesized under different aging temperature (from room temperature to 140°C) impregnated with PEI.

**Sample**	**S_BET_ (m^2^g^−1^)**	**t-plot (m^2^g^−1^)**	**N (wt.%)**	**q_15_ [mmol g^−1^]**	**q_m2_ [mmol g^−1^]**	**mol CO_2_/mol N (q_m2_)**
MCF-RT 50P	29	10	14.206	0.357	0.339	0.033
MCF-60 50P	43	12	13.240	1.481	1.523	0.161
MCF-80 50P	59	19	14.651	1.696	1.676	0.160
MCF-100 50P	69	28	13.824	1.942	1.953	0.198
MCF-120 50P	65	25	13.035	1.845	1.712	0.184
MCF-140 50P	62	23	14.676	1.839	1.788	0.171

*q_15_, quantity of CO_2_ adsorbed at 15 kPa*.

The comparison between SBA-15 and MCF aged at 100°C (Supplementary Figure 6) reveals that the impregnation with 50 wt.% PEI leads to higher CO_2_ adsorption capacity in the case of the MCF. MCF has higher pore diameter and shorter channels, which favors the pore filling with PEI molecules and enhances diffusion in such a way that a larger amount of available amine sites is involved in CO_2_ chemisorption.

#### Functionalization of the MCFs by Impregnation With TEPA

The MFCs were also functionalized by impregnation with another amine rich polymer, TEPA. The FT-IR spectrum of MCF-100 50T shows a similar profile to the case of MCF-100 50P, while the elemental CHN analyses of the adsorbents impregnated with TEPA are in the same range as measured for MCFs impregnated with PEI (11.042-14.676 wt.%).

**Figure 8:**
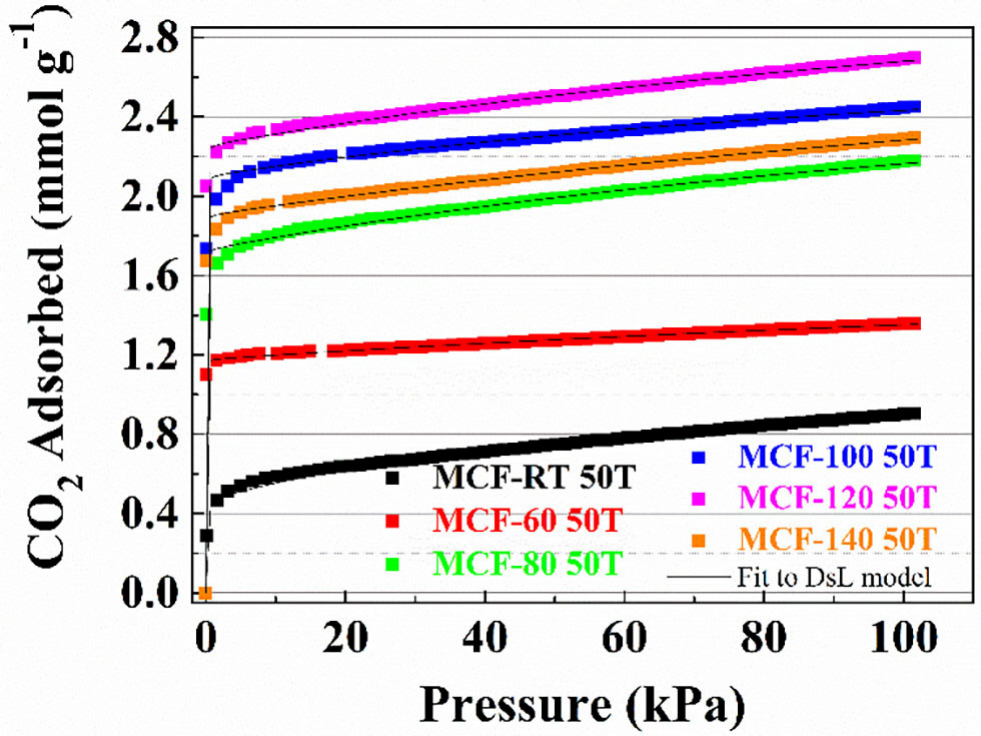
CO_2_ adsorption isotherms at 25°C of the porous silicas (MCFs) synthesized under different aging temperature (from room temperature to 140°C) impregnated with TEPA. Solid lines for fit to DsL model.

The CO_2_ adsorption isotherms for TEPA-impregnated MCFs are compiled in Figure 8. Similarly to those samples impregnated with PEI (Figure 7), all isotherms show a clearly rectangular profile, indicating that the adsorption process is also eminently governed by chemisorption. It is noteworthy that CO_2_ uptake improves as the aging temperature increases: from 0.89 mmol CO_2_ g^−1^ for MCF-RT 50T to 2.70 mmol CO_2_ g^−1^ for MCF-120 50T, which is directly related to the larger pore diameter and pore volume (Figure 3B and Table 2). Despite the similarities with PEI-impregnated samples (Figure 7), CO_2_ adsorption in TEPA-impregnated MCFs is higher because TEPA is a less bulky molecule with lower viscosity than PEI. These features are likely to lessen the blocking effect of the amine-rich molecule on the surface of the adsorbent and its pores, which favors the availability of CO_2_ chemisorption sites.

The adsorption isotherms were fitted to the Dualsite Langmuir model (Table 9). As observed for the PEI-impregnated MCFs, the higher value of the parameter b for the chemical sites (b_2_) confirms the stronger adsorbate-adsorbent interactions. The efficiency of the TEPA-impregnated samples (Table 10) shows CO_2_/N molar ratios that are slightly higher than those reported by the PEI-impregnated MCFs, confirming that the N-species of the TEPA are more available and therefore more efficient than in the case of PEI-impregnated MCFs. This fact leads to a higher CO_2_ adsorption capacity.

**Table 9:** Dualsite Langmuir model fitting parameters for MCFs impregnated with TEPA.

**Sample**	**q_m1_ (mmolg^−1^)**	b_1_ *10^3^ (kPa^−1^)	**q_m2_ (mmolg^−1^)**	**b_2_ (kPa^−1^)**	**ARE (%)**
MCF-RT-50T	0.698	1.933	0.465	4.932	1.969
MCF-60-50T	0.745	3.064	1.177	227.988	0.191
MCF-80-50T	1.265	5.217	1.731	226.052	0.638
MCF-100-50T	0.855	6.358	2.101	203.849	0.582
MCF-120-50T	1.332	4.684	2.254	202.114	0.328
MCF-140-50T	1.502	3.376	1.904	202.117	0.307

**Table 10:** Textural properties, nitrogen content and efficiency of the N-sites for the MCFs synthesized under different aging temperature (from room temperature to 140°C) impregnated with TEPA.

**Sample**	**S_BET_ (m^2^g^−1^)**	**t-plot (m^2^g^−1^)**	**N (wt.%)**	**q_15_ [mmol g^−1^]**	**q_m2_ (mmolg−1)**	**mol CO_2_/mol N (q_15_)**
MCF-RT 50T	21	8	14.676	0.604	0.465	0.044
MCF-60 50T	40	10	12.055	1.211	1.177	0.137
MCF-80 50T	62	21	12.838	1.839	1.731	0.189
MCF-100 50T	73	31	11.042	2.181	2.101	0.266
MCF-120 50T	79	34	14.104	2.376	2.254	0.224
MCF-140 50T	72	29	11.056	1.986	1.904	0.241

*q_15_, quantity of CO_2_ adsorbed at 15 kPa*.

The comparison between SBA-15 and MCF aged at the same temperature (100°C) (Supplementary Figure 7) shows how functionalization by impregnation with TEPA is more efficient for MCF. As explained previously, the higher pore diameter distribution and pore volume of the MCF enhances the dispersion of the amine sites. This feature is less pronounced than that observed for PEI, probably because TEPA is a less bulky polymer. As a result, TEPA is more evenly dispersed in the adsorbent pores in comparison to PEI, which seems to provide an enhanced availability of chemical adsorption sites.

### Influence of the Temperature in the CO_2_ Adsorption Capacity

**Figure 9:**
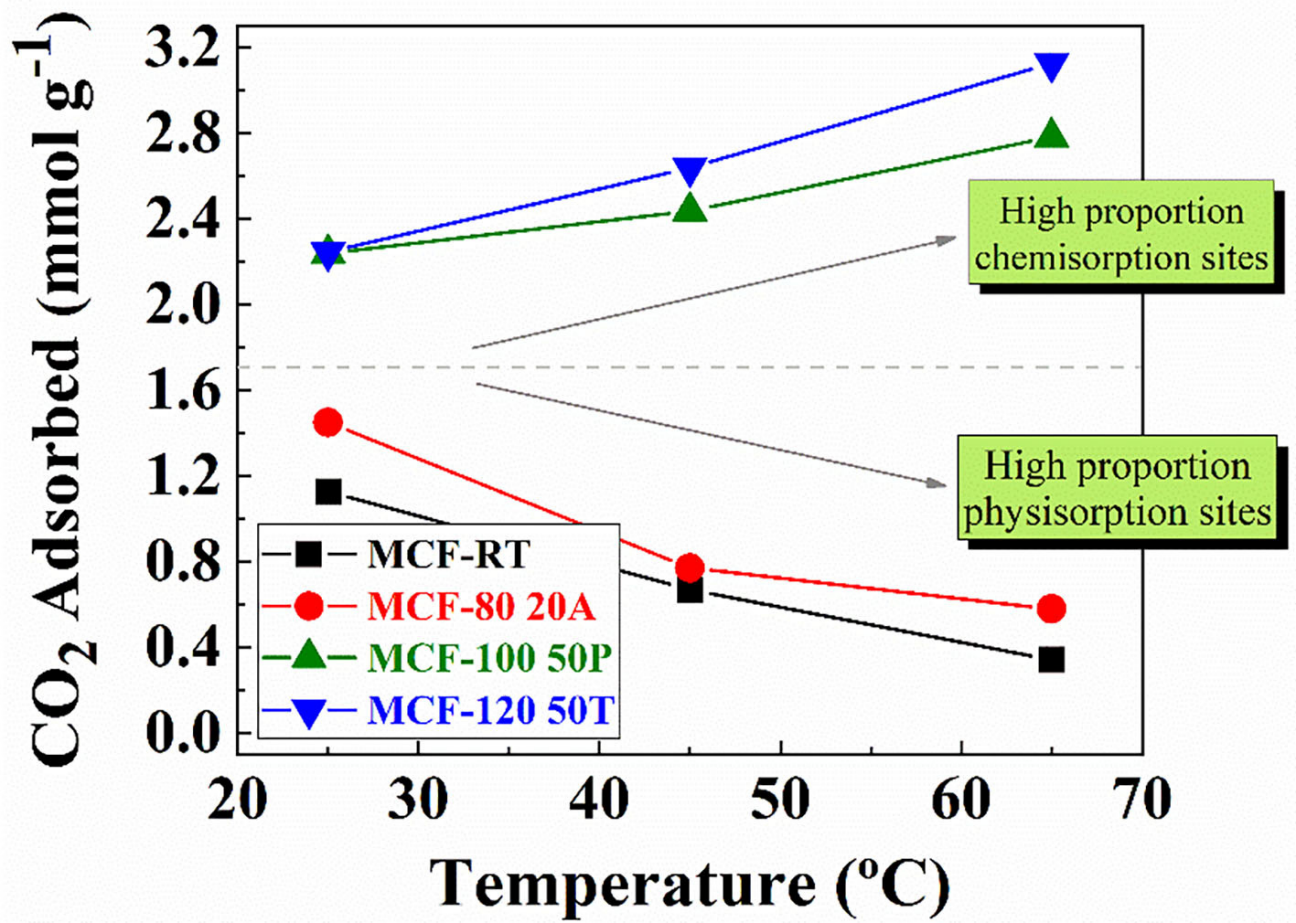
CO_2_ amount adsorbed for MCF-RT, MCF80-20A, MCF100-50P, and MCF120-50T at 25, 45, and 65°C and 100 kPa.

The adsorbents with the highest CO_2_ adsorption capacity of each set of adsorbents, that is, MCF-RT, MCF-80-20A, MCF-100-50P, and MCF-120-50T, were chosen to carry out experiments under at different adsorption temperatures. In the case of the MCF-RT and MCF-80-20A samples (Figure 9), increasing the adsorption temperature causes a drastic decrease in the CO_2_ uptake (at 100 kPa): from 1.13 mmol CO_2_ g^−1^ at 25°C to 0.41 mmol CO_2_ g^−1^ at 65°C for pure MCF-RT and from 1.45 mmol CO_2_ g^−1^ at 25°C to 0.66 mmol CO_2_ g^−1^ at 65°C for MCF-80 20A. In the case of MCF-RT, this drop is attributed to adsorption being mainly governed by physical interactions (Do, 1998; Rouquerol et al., 2014; Thommes et al., 2015), which are favored at lower temperatures. The sample functionalized with APTES with highest CO_2_ adsorption capacity (MCF-80-20A) (Figure 9) follows a similar trend as that observed for the purely physical adsorbent, uptakes decreasing with increasing temperature. The presence of amine groups should favor CO_2_ chemisorption, which is likely to benefit from temperature increases (Vilarrasa-García et al., 2015b, 2017a; Cecilia et al., 2016). However, the observed behavior for the APTES-grafted MCF suggests that grafting covered only a fraction of the surface, while keeping the porous character responsible for physical adsorption, which is unfavored at higher temperatures. This is in agreement with the low concentration of silanol groups after calcination which limited the amount of fixed nitrogen to no more than 3.3% wt and with the low amount of CO_2_ chemisorbed over CO_2_ physisorbed ratio, as it shown in Supplementary Table 1. Therefore, despite the appearance of chemisorption sites, adsorption is likely to be predominantly governed by physisorption in APTES-grafted MCF samples.

The adsorbents impregnated with PEI and TEPA that showed the highest CO_2_ uptakes were MCF-100-50P and MCF-120-50T. At higher temperatures, CO_2_ adsorption capacity is improved for both samples, reaching a maximum value of 2.91 mmol CO_2_ g^−1^ at 65°C for MCF-100 50P and 3.24 mmol CO_2_ g^−1^ at 65°C for MCF-120 50T. In this sense, previous authors have reported that porous silicas impregnated with amine rich-polymer improve their CO_2_ adsorption capacity as a consequence of a re arrangement of the polymer stacking that increases the amount of available N-species and minimizes diffusion problems in the porous structure (Xu et al., 2002; Wang et al., 2009, 2013; Sanz et al., 2010; Vilarrasa-García et al., 2017b).

### Stability of the Adsorbents

**Figure 10:**
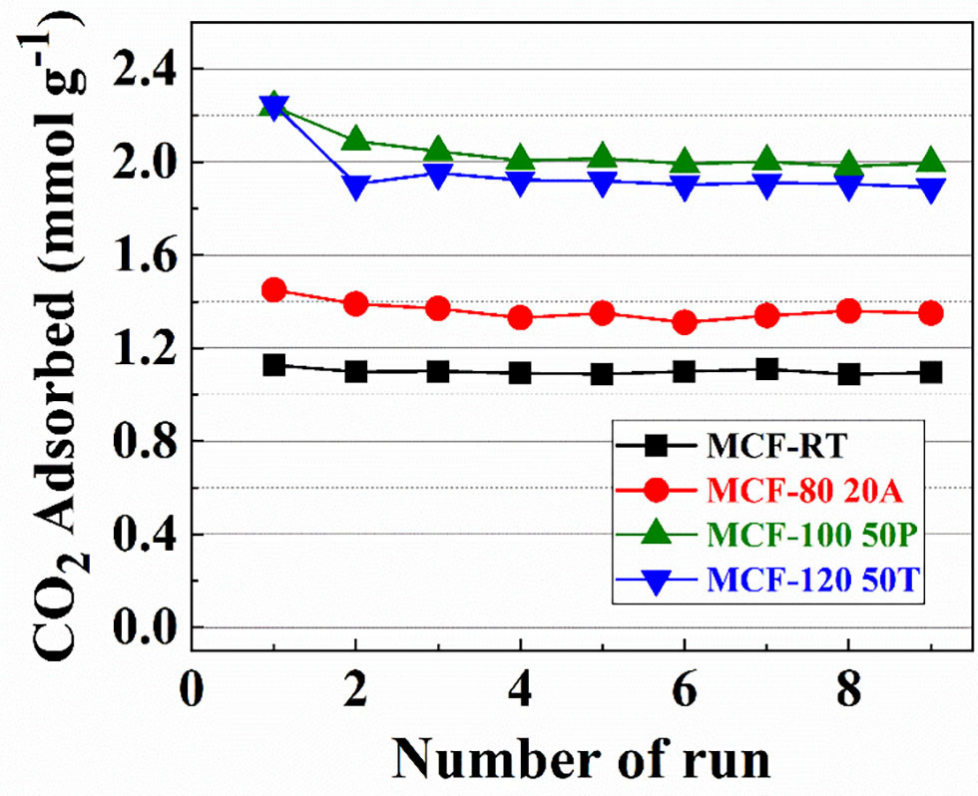
CO_2_ amount adsorbed for MCF-RT, MCF80-20A, MCF100-50P, and MCF120-50T at 25°C and 100 kPa for 9 runs.

In order to assess the stability of the samples with the highest CO_2_ adsorption capacity of each set of adsorbents (MCF-RT, MCF-80-20A, MCF-100-50P, and MCF-120-50T), several CO_2_ adsorption/desorption runs were carried out (Figure 10).

Nearly all of the samples are stable after nine cycles. The sample grafted with APTES suffers a slight decrease after several runs, decreasing from 1.444 to 1.354 mmol CO_2_ g^−1^. In the case of the samples impregnated with PEI or TEPA this decrease is more pronounced, particularly in the first runs. This is probably due to the regeneration conditions (110°C and 10^−4^ mbar), which can remove a small proportion of N-species that are not sufficiently bound to the support by electrostatic interactions.

## Conclusions

The use of structure modifying agents and varying aging temperature were successful strategies to obtain silica mesocellular foams with increasing pore diameter. APTES-grafting of these structures enhances CO_2_ adsorption, even though the amount of loaded amino groups is restricted by the low density of silanols on the surface. Despite the chemical interaction brought about by grafted primary amines from APTES, physisorption has a leading role over chemisorption in these MCFs and increasing temperature has an adverse effect on CO_2_ uptakes.

In the case of samples impregnated with PEI and TEPA, CO_2_ adsorption is mainly attributed to the chemical interaction between the amines species and the CO_2_. CO_2_/N molar ratios are considerably lower than those obtained for APTES-grafted due to less availability of amino groups, despite the higher N-content made possible by pore filling with the amine polymer. This feature is less pronounced in the case of TEPA due to its lower viscosity and molar volume as compared to PEI. Unlike APTES-grafted MCFs, for samples impregnated with PEI or TEPA, chemisorption governs CO_2_ retention over physisorption. As a result, CO_2_ uptakes are improved with increasing temperature.

All functionalized samples showed a relatively stable uptake for up to nine adsorption-desorption runs, in which case CO_2_ was desorbed by increasing the temperature and lowering the pressure. The amine-functionalized materials, particularly TEPA-impregnated MCFs, show potential for CO_2_ capture from dilute and moderately hot streams. Under these typical post-combustion conditions (15 kPa and 65°C), they may reach the competitive adsorbed concentrations of 3 mmol/g and be cyclically used under moderate temperature swings.

## Data Availability Statement

The raw data supporting the conclusions of this article will be made available by the authors, without undue reservation.

## Author Contributions

EV-G: participated in redaction of the manuscript and in the discussion of results. JC: participated in the synthesis and characterization of the materials and in the discussion of results. PM: participated in the discussion of results. DA: participated in all the research. ER-C: participated in all the research. The manuscript was written through contributions of all authors and have given approval to the final version of the manuscript.

## Conflict of Interest

The authors declare that the research was conducted in the absence of any commercial or financial relationships that could be construed as a potential conflict of interest.

## Supplementary Material

Supplementary Figure 1 Comparison between SAXS profiles of MCF and SBA-15 aging at 100°C.

Supplementary Figure 2 Comparison of the N2 adsorption-desorption isotherms at -196°C between MCF and SBA-15 synthesized at aging temperature 100°C.

Supplementary Figure 3 Comparison of pore size distributions determined by NLDFT method between MCF and SBA-15 synthesized at aging temperature 100°C.

Supplementary Figure 4 Comparison of CO_2_ adsorption isotherms at 25°C between MCF and SBA-15 aging at 100°C.

Supplementary Figure 5 Comparison of CO_2_ adsorption isotherms at 25°C between MCF and SBA-15 aging at 100°C grafted with APTES.

Supplementary Figure 6 Comparison of CO_2_ adsorption isotherms at 25°C between MCF and SBA-15 aging at 100°C impregnated with PEI.

Supplementary Figure 7 Comparison of CO_2_ adsorption isotherms at 25°C between MCF and SBA-15 aging at 100°C impregnated with TEPA.

Supplementary Table 1 Relation between amount of CO_2_ adsorbed by chemisorption and amount of CO_2_ adsorbed by physisorption at 25°C calculated from the fitting parameters of the Dualsite Langmuir model.

## References

[B1] Alkhabbaz M. A. Bollini P. Foo G. S. Sievers C. Jones C. W. (2014). Important roles of enthalpic and entropic contributions to CO_2_ capture from simulated flue gas and ambient air using mesoporous silica grafted amines. J. Am. Chem. Soc. 136, 13170–13173. 10.1021/ja507655x 25215519

[B2] Ballesteros-Plata D. Infantes-Molina A. Rodríguez-Castellón E. (2019). Study of bifunctionality of Pt/SBA-15 catalysts for HDO of Dibenzofuran reaction: addition of Mo or use of an acidic support. Appl. Catal. A Gen. 580, 93–101. 10.1016/j.apcata.2019.05.002

[B3] Beck J. S. Vartuli J. C. Roth W. J. Leonowicz M. E. Kresge C. T. Schmitt K. D. (1992). A new family of mesoporous molecular sieves prepared with liquid crystal templates. J. Am. Chem. Soc. 114, 10834–10843. 10.1021/ja00053a020

[B4] Bezerra D. P. da Silva F. W. M. Moura P. A. S. Sousa A. G. S. Vieira RS Rodriguez-Castellon E (2014). CO_2_ adsorption in amine-grafted zeolite 13X. Appl. Surf. Sci. 314, 314–321. 10.1016/j.apsusc.2014.06.164

[B5] Bishnoi S. Rochelle G. T. (2000). Absorption of carbon dioxide into aqueous piperazine: reaction kinetics, mass transfer and solubility. Chem. Eng. Sci. 55, 5531–5543. 10.1016/S0009-2509(00)00182-2

[B6] Borodko Y. Ager J. W. Marti G. E. Song H. Niesz K. Somorjai G. A. (2005). Structure sensitivity of vibrational spectra of mesoporous silica SBA-15 and Pt/SBA-15. J. Phys. Chem. B. 109, 17386–17390. 10.1021/jp051801x 16853222

[B7] Brunauer S. Emmett P. H. Teller E. (1938). Adsorption of gases in multimolecular layers. J. Am. Chem. Soc. 60, 309–319. 10.1021/ja01269a023

[B8] Burneau A. Barres O. Gallas J. P. Lavalley J. C. (1990). Comparative study of the surface hydroxyl groups of fumed and precipitated silicas. 2. Characterization by infrared spectroscopy of the interactions with water. Langmuir 6, 1364–1372. 10.1021/la00098a008

[B9] Caplow M. (1968). Kinetics of carbamate formation and breakdown. J. Am. Chem. Soc. 24, 6795–6803. 10.1021/ja01026a041

[B10] Cecilia J. A. Vilarrasa-García E. Cavalcante C. L. Azevedo D. C. S. Franco F. Rodríguez-Castellón E. (2018). Evaluation of two fibrous clay minerals (sepiolite and palygorskite) for CO_2_ capture. J. Environ. Chem. Eng. 6, 4573–4587. 10.1016/j.jece.2018.07.001

[B11] Cecilia J. A. Vilarrasa-García E. García-Sancho C. Saboya R. M. A. Azevedo D. C. S. Cavalcante C. L. (2016). Functionalization of hollow silica microspheres by impregnation or grafted of amine groups for the CO_2_ capture. Int. J. Greenhouse Gas Control. 52, 344–356. 10.1016/j.ijggc.2016.07.018

[B12] Cecilia J. A. Vilarrasa-García E. Morales-Ospino R. Bastos-Neto M. Azevedo D. C. S. Rodríguez-Castellón E. (2020). Insights into CO_2_ adsorption in amino-functionalized SBA-15 synthesized at different aging temperature. Adsorption 26, 225–240. 10.1007/s10450-019-00118-1 PMC7702615 33313041

[B13] Chen C. Lee Y.-R. Ahn W.-S. (2016). CO_2_ adsorption over metal-organic frameworks: a mini review. J. Nanosci. Nanotechnol. 16, 4291–4301. 10.1166/jnn.2016.10971 27483749

[B14] Chen Q. Fan F. Long D. Liu X. Liang X. Qiao W. (2010). Poly(ethyleneimine-loaded silica monolith with hierarchical pore structure for H_2_S adsorptiove removal. Ind. Eng. Chem. Res. 49, 11408–11414. 10.1021/ie101464f

[B15] Choi S. Drese J. H. Jones C. W. (2009). Adsorbent materials for carbon dioxide capture from large anthropogenic point sources. ChemSusChem 2, 796–854. 10.1002/cssc.200900036 19731282

[B16] Colilla M. Izquierdo-Barba I. Sánchez-Salcedo S. Fierro J. L. G. Hueso J. L. Vallet-Reg,í M. (2010). Synthesis and characterization of Zwitterionic SBA-15 nanostructured materials. Chem. Mater. 22, 6459–6466. 10.1021/cm102827y

[B17] Danckwerts P. V. (1979). The reaction of CO_2_ with ethanolamines. Chem. Eng. Sci. 34, 443–446. 10.1016/0009-2509(79)85087-3

[B18] de Boer J. H. Lippens B. C. Linsen B. G. Broekhoff J. C. P. van den Heuvel A. Osinga T. J. (1966). Thet-curve of multimolecular N_2_-adsorption. J. Colloid Interface Sci. 21, 405–414. 10.1016/0095-8522(66)90006-7

[B19] Didas S. A. Sakwa-Novak M. A. Foo G. S. Sievers C. Jones C. W. (2014). Effect of amine surface coverage on the co-adsorption of CO_2_ and water: spectral deconvolution of adsorbed species. J. Phys. Chem. Lett. 5, 4194–4200. 10.1021/jz502032c 26278953

[B20] Do D. D. (1998). Adsorption Analysis : Equilibria and Kinetics. London, UK: Imperial College Press 10.1142/p111

[B21] Donaldson T. L. Nguyen Y. N. (1980). Carbon dioxide reaction kinetics and transport in aqueous amine membranes. Ind. Eng. Chem. Fundament. 19, 260–266. 10.1021/i160075a005

[B22] Feng X. Hu G. Hu X. Xie G. Xie Y. Lu J. (2013). Tetraethylenepentamine-Modified Siliceous Mesocellular Foam (MCF) for CO_2_ Capture. Ind. Eng. Chem. Res. 52, 4221–4228. 10.1021/ie301946p

[B23] Galarneau A. Cambon H. Di Renzo F. Fajula F. (2001). True microporosity and surface area of mesoporous SBA-15 silicas as a function of synthesis temperature. Langmuir 17, 8328–8335. 10.1021/la0105477

[B24] Gargiulo N. Pepe F. Caputo D. (2014). CO_2_ Adsorption by functionalized nanoporous materials: a review. J. Nanosci. Nanotechnol. 14, 1811–1822. 10.1166/jnn.2014.8893 24749458

[B25] Guo B. Chang L. Xie K. (2006). Adsorption of carbon dioxide on activated carbon. J. Nat. Gas Chem. 15, 223–229. 10.1016/S1003-9953(06)60030-320407992

[B26] Hanif A. Dasgupta S. Divekar S. Arya A. Garg M. O. Nanoti A. (2014). A study on high temperature CO2 capture by improved hydrotalcite sorbents. Chem. Eng. J. 236, 91–99. 10.1016/j.cej.2013.09.076

[B27] Hiremath V. Shavi R. Gil Seo J. (2017). Mesoporous magnesium oxide nanoparticles derived via complexation-combustion for enhanced performance in carbon dioxide capture. J. Colloid Interface Sci. 498, 55–63. 10.1016/j.jcis.2017.03.046 28319841

[B28] Hiyoshi N. Yogo K. Yashima T. (2004). Adsorption of carbon dioxide on modified mesoporous materials in the presence of water vapor. Stud. Surf. Sci. Catal. 154, 2995–3002. 10.1016/S0167-2991(04)80583-4

[B29] Hiyoshi N. Yogo K. Yashima T. (2005). Adsorption of carbon dioxide on aminosilane-modified mesoporous silica. J. Japan Petrol. Inst. 48, 29–36. 10.1627/jpi.48.29

[B30] Hu Y. Liu X. Zhou Z. Liu W. Xu M. (2017). Pelletization of MgO-based sorbents for intermediate temperature CO_2_ capture. Fuel 187, 328–337. 10.1016/j.fuel.2016.09.066

[B31] Hwang C.-C. Tour J. J. Kittrell C. Espinal L. Alemany L. B. Tour J. M. (2014). Capturing carbon dioxide as a polymer from natural gas. Nat. Commun. 5:3961. 10.1038/ncomms4961 24892923 PMC5603724

[B32] Intergovernmental Panel on Climate Change. Working Group III Edenhofer O. (2014). Climate Change 2014 : Mitigation of Climate Change : Working Group III Contribution to the Fifth Assessment Report of the Intergovernmental Panel on Climate Change. New York, NY: Cambridge University Press 10.1017/CBO9781107415416

[B33] Ko Y. G. Shin S. S. Choi U. S. (2011). Primary, secondary, and tertiary amines for CO_2_ capture: designing for mesoporous CO_2_ adsorbents. J. Colloid Interface Sci. 136, 594–602. 10.1016/j.jcis.2011.03.045 21708387

[B34] Kruk M. Cao L. (2007). Pore size tailoring in large-pore SBA-15 silica synthesized in the presence of hexane. Langmuir 23, 7247–7254. 10.1021/la0702178 17503860

[B35] Landers J. Gor G. Y. Neimark A. V. (2013). Density functional theory methods for characterization of porous materials. Colloids Surf. A Physicochem. Eng. Aspects 437, 3–32. 10.1016/j.colsurfa.2013.01.007

[B36] Langmuir I. (1918). The adsorption of gases on plane surfaces of glass, mica and platinum. J. Am. Chem. Soc. 40, 1361–1403. 10.1021/ja02242a004

[B37] Lettow J. S. Han Y. J. Schmidt-Winkel P. Yang P. Zhao D. Stucky G. D. (2000). Hexagonal to mesocellular foam phase transition in polymer-templated mesoporous silicas. Langmuir 16, 8291–8295. 10.1021/la000660h

[B38] Luo C. Zheng Y. Xu Y. Ding N. Shen Q. Zheng C. (2015). Wet mixing combustion synthesis of CaO-based sorbents for high temperature cyclic CO2 capture. Chem. Eng. J. 267, 111–116. 10.1016/j.cej.2015.01.005

[B39] Ma'mun S. Svendsen H. F. Hoff K. A. Juliussen O. (2007). Selection of new absorbents for carbon dioxide capture. Energy Convers. Manage. 48, 251–258. 10.1016/j.enconman.2006.04.007

[B40] Mebane D. S. Kress J. D. Storlie C. B. Fauth D. J. Gray M. L. Li K. (2013). Transport, Zwitterions, and the role of water for CO_2_ adsorption in mesoporous silica-supported amine sorbents. J. Phys. Chem. C 117, 26617–26627. 10.1021/jp4076417

[B41] Moura P. A. S. Vilarrasa-Garcia E. Maia D. A. S. Bastos-Neto M. Ania C. O. Parra J. B. (2018). Assessing the potential of nanoporous carbon adsorbents from polyethylene terephthalate (PET) to separate CO_2_ from flue gas. Adsorption 24, 279–291. 10.1007/s10450-018-9943-4

[B42] Nguyen T. P. B. Lee J.-W. Shim W. G. Moon H. (2008). Synthesis of functionalized SBA-15 with ordered large pore size and its adsorption properties of BSA. Microporous Mesoporous Mater. 110, 560–569. 10.1016/j.micromeso.2007.06.054

[B43] Oyenekan B. A. Rochelle G. T. (2007). Alternative stripper configurations for CO_2_ capture by aqueous amines. AIChE J. 53, 3144–3154. 10.1002/aic.11316

[B44] Pera-Titus M. (2014). Porous inorganic membranes for CO_2_ capture: present and prospects. Chem. Rev. 114, 1413–1492. 10.1021/cr400237k 24299113

[B45] Pinto M. L. Mafra L. Guil J. M. Pires J. Rocha J. (2011). Adsorption and activation of CO_2_ by amine-modified nanoporous materials studied by solid-state NMR and ^13^CO_2_ adsorption. Chem. Mater. 23, 1387–1395. 10.1021/cm1029563

[B46] Potter M. E. Pang S. H. Jones C. W. (2017). Adsorption microcalorimetry of CO_2_ in confined aminopolymers. Langmuir 33, 117–124. 10.1021/acs.langmuir.6b03793 27992227

[B47] Ridha F. N. Manovic V. Macchi A. Anthony E. J. (2015). CO_2_ capture at ambient temperature in a fixed bed with CaO-based sorbents. Appl. Energy 140, 297–303. 10.1016/j.apenergy.2014.11.030

[B48] Rouquerol F. Rouquerol J. Sing K. S. W. Llewellyn P. L. Maurin G. (2014). Adsorption by Powders and Porous Solids : Principles, Methodology and Applications. 2nd ed Amsterdam: Elsevier/AP.

[B49] Samanta A. Zhao A. Shimizu G. K. H. Sarkar P. Gupta R. (2012). Post-combustion CO_2_ capture using solid sorbents: a review. Ind. Eng. Chem. Res. 51, 1438–1463. 10.1021/ie200686q

[B50] Sánchez-Zambrano K. S. Vilarrasa-García E. Maia D. A. S. Bastos-Neto M. Rodríguez-Castellon E. Azevedo D. C. S. (2020). Adsorption microcalorimetry as a tool in the characterization of amine-grafted mesoporous silicas for CO_2_ capture. Adsorption 26, 165–175. 10.1007/s10450-019-00064-y

[B51] Santos S. M. L. Cecilia J. A. Vilarrasa-García E. Silva Junior I. J. Rodríguez-Castellón E. Azevedo D. C. S. (2016). The effect of structure modifying agents in the SBA-15 for its application in the biomolecules adsorption. Microporous Mesoporous Mater. 232, 53–64. 10.1016/j.micromeso.2016.06.004

[B52] Sanz R. Calleja G. Arencibia A. Sanz-Pérez E. S. (2010). CO_2_ adsorption on branched polyethyleneimine-impregnated mesoporous silica SBA-15. Appl. Surf. Sci. 256, 5323–5328. 10.1016/j.apsusc.2009.12.070

[B53] Sanz-Pérez E. S. Murdock C. R. Didas S. A. Jones C. W. (2016). Direct capture of CO_2_ from ambient air. Chem. Rev. 116, 11840–11876. 10.1021/acs.chemrev.6b00173 27560307

[B54] Sayari A. Belmabkhout Y. Serna-Guerrero R. (2011). Flue gas treatment via CO_2_ adsorption. Chem. Eng. J. 171, 760–774. 10.1016/j.cej.2011.02.007

[B55] Sayari A. Kruk M. Jaroniec M. Moudrakovski I. L. (1998). New approaches to pore size engineering of mesoporous silicates. Adv. Mater. 10, 1376–1379. 10.1002/(SICI)1521-4095(199811)10:16<1376::AID-ADMA1376>3.0.CO;2-B

[B56] Schmidt-Winkel P. Lukens W. W. Yang P. Margolese D. I. Lettow J. S. Ying J. Y. (2000). Microemulsion templating of siliceous mesostructured cellular foams with well-defined ultralarge mesopores. Chem. Mater. 12, 686–696. 10.1021/cm991097v

[B57] Serna-Guerrero R. Belmabkhout Y. Sayari A. (2010). Modeling CO_2_ adsorption on amine-functionalized mesoporous silica: 1. A semi-empirical equilibrium model. Chem. Eng. J. 161, 173–181. 10.1016/j.cej.2010.04.024

[B58] Serreze M. C. (2010). Understanding recent climate change. Conserv. Biol. 24, 10–17. 10.1111/j.1523-1739.2009.01408.x 20121837

[B59] Silva J. M. Trujillano R. Rives V. Soria M. A. Madeira L. M. (2017). High temperature CO_2_ sorption over modified hydrotalcites. Chem. Eng. J. 325, 25–34. 10.1016/j.cej.2017.05.032

[B60] Singh S. Kumar R. Setiabudi H. D. Nanda S. Vo D.-V. N. (2018). Advanced synthesis strategies of mesoporous SBA-15 supported catalysts for catalytic reforming applications: a state-of-the-art review. Appl. Catal. A Gen. 559, 57–74. 10.1016/j.apcata.2018.04.015

[B61] Siriwardane R. V. Shen M.-S. Fisher E. P. Losch J. (2005). Adsorption of CO_2_ on zeolites at moderate temperatures. Energy Fuels 19, 1153–1159. 10.1021/ef040059h

[B62] Sumida K. Rogow D. L. Mason J. A. McDonald T. M. Bloch E. D. Herm Z. R.. (2012). Carbon dioxide capture in metal–organic frameworks. Chem. Rev. 112, 724–781. 10.1021/cr2003272 22204561

[B63] Thommes M. Kaneko K. Neimark A. V. Olivier J. P. Rodriguez-Reinoso F. Rouquerol J. (2015). Physisorption of gases, with special reference to the evaluation of surface area and pore size distribution (IUPAC Technical Report). Pure Appl. Chem. 87, 1051–1069. 10.1515/pac-2014-1117

[B64] Vilarrasa-García E. Cecilia A. J. Moya M. E. Cavalcante L. C. Azevedo C. D. Rodríguez-Castellón E. (2015a). “Low cost” pore expanded SBA-15 functionalized with amine groups applied to CO_2_ adsorption. Materials 8, 2495–2513. 10.3390/ma8052495

[B65] Vilarrasa-García E. Cecilia J. A. Bastos-Neto M. Cavalcante C. L. Azevedo D. C. S. Rodríguez-Castellón E. (2017a). Microwave-assisted nitric acid treatment of sepiolite and functionalization with polyethylenimine applied to CO_2_ capture and CO_2_/N_2_ separation. Appl. Surf Sci. 410, 315–325. 10.1016/j.apsusc.2017.03.054

[B66] Vilarrasa-García E. Cecilia J. A. Rodríguez Aguado E. Jiménez-Jiménez J. Cavalcante C. L. Azevedo D. C. S. (2017b). Amino-modified pillared adsorbent from water-treatment solid wastes applied to CO_2_/N_2_ separation. Adsorption 23, 405–421. 10.1007/s10450-017-9871-8

[B67] Vilarrasa-García E. Cecilia J. A. Santos S. M. L. Cavalcante C. L. Jiménez-Jiménez J. Azevedo D. C. S. (2014). CO_2_ adsorption on APTES functionalized mesocellular foams obtained from mesoporous silicas. Microporous Mesoporous Mater. 187, 125–134. 10.1016/j.micromeso.2013.12.023

[B68] Vilarrasa-García E. Moya E. M. O. Cecilia J. A. Cavalcante C. L. Jiménez-Jiménez J. Azevedo D. C. S. (2015b). CO_2_ adsorption on amine modified mesoporous silicas: effect of the progressive disorder of the honeycomb arrangement. Microporous Mesoporous Mater. 209, 172–183. 10.1016/j.micromeso.2014.08.032

[B69] Wang J. Huang L. Yang R. Zhang Z. Wu J. Gao Y. (2014). Recent advances in solid sorbents for CO_2_ capture and new development trends. Energy Environ. Sci. 7, 3478–3518. 10.1039/C4EE01647E

[B70] Wang X. Lin K. S. K. Chan J. C. C. Cheng S. (2005). Direct synthesis and catalytic applications of ordered large pore aminopropyl-functionalized SBA-15 mesoporous materials. J. Phys. Chem. B 109, 1763–1769. 10.1021/jp045798d 16851156

[B71] Wang X. Ma X. Song C. Locke D. R. Siefert S. Winans R. E.. (2013). Molecular basket sorbents polyethylenimine–SBA-15 for CO_2_ capture from flue gas: characterization and sorption properties. Microporous Mesoporous Mater. 169, 103–111. 10.1016/j.micromeso.2012.09.02328940634

[B72] Wang X. Schwartz V. Clark J. C. Ma X. Overbury S. H. Xu X. (2009). Infrared study of CO_2_ sorption over “molecular basket” sorbent consisting of polyethylenimine-modified mesoporous molecular sieve. J. Phys. Chem. C 113, 7260–7268. 10.1021/jp809946y

[B73] Wickramaratne N. P. Jaroniec M. (2013). Activated carbon spheres for CO_2_ adsorption. ACS Appl. Mater. Interfaces 5, 1849–1855. 10.1021/am400112m 23398600

[B74] Xu X. Song C. Andresen J. M. Miller B. G. Scaroni A. W. (2002). Novel polyethylenimine-modified mesoporous molecular sieve of MCM-41 type as high-capacity adsorbent for CO_2_ capture. Energy Fuels 16, 1463–1469. 10.1021/ef020058u

[B75] Yao B. Fleming D. Morris M. A. Lawrence S. E. (2004). Structural control of mesoporous silica nanowire arrays in porous alumina membranes. Chem. Mater. 16, 4851–4855. 10.1021/cm0487425

