# Supplementary material for: Assessing CO2 Adsorption on Amino-Functionalized Mesocellular Foams Synthesized at Different Aging Temperatures
Source: Front Chem. 2020 Nov 16;8:591766. doi: 10.3389/fchem.2020.591766 (PMC7702615; doi:10.3389/fchem.2020.591766)
Supplement: Supplementary Figure 1 — Comparison between SAXS profiles of MCF and SBA-15 aging at 100°C. [file Data_Sheet_1.docx]

| 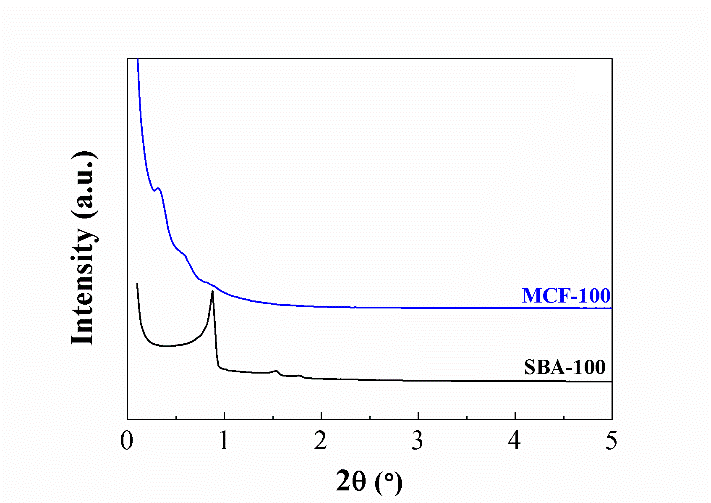 |
| --- |
| **Figure S1.** Comparison between SAXS profiles of MCF and SBA-15 aging at 100 ºC. |

| 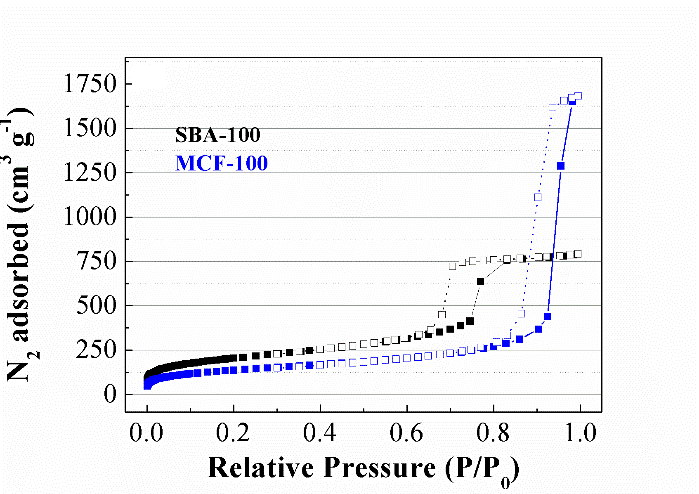 |
| --- |
| **Figure S2.** Comparison of the N_2_ adsorption-desorption isotherms at -196ºC between MCF and SBA-15 synthesized at aging temperature 100 ºC. |

| 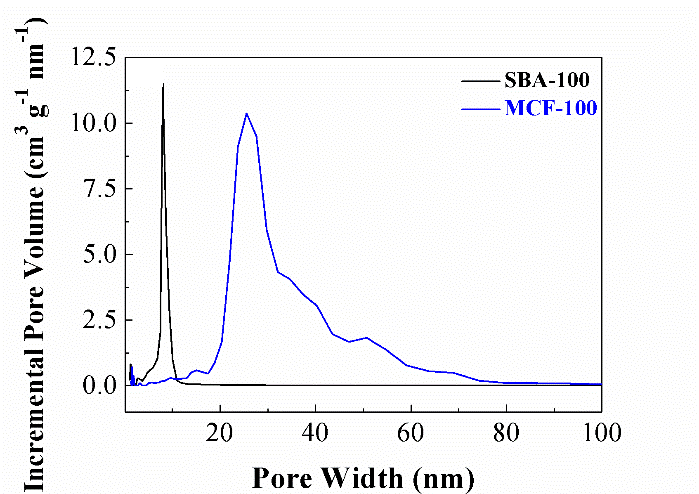 |
| --- |
| **Figure S3.** Comparison of pore size distributions determined by NLDFT method between MCF and SBA-15 synthesized at aging temperature 100 ºC. |

|  |
| --- |
| **Figure S4.** Comparison of CO_2_ adsorption isotherms at 25ºC between MCF and SBA-15 aging at 100 ºC. |

|  |
| --- |
| **Figure S5.** Comparison of CO_2_ adsorption isotherms at 25ºC between MCF and SBA-15 aging at 100 ºC grafted with APTES. |

|  |
| --- |
| **Figure S6.** Comparison of CO_2_ adsorption isotherms at 25ºC between MCF and SBA-15 aging at 100 ºC impregnated with PEI. |

|  |
| --- |
| **Figure S7.** Comparison of CO_2_ adsorption isotherms at 25ºC between MCF and SBA-15 aging at 100 ºC impregnated with TEPA. |
