# Supplementary material for: Assessing CO2 Adsorption on Amino-Functionalized Mesocellular Foams Synthesized at Different Aging Temperatures
Source: Front Chem. 2020 Nov 16;8:591766. doi: 10.3389/fchem.2020.591766 (PMC7702615; doi:10.3389/fchem.2020.591766)
Supplement: Supplementary Table 1 — Relation between amount of CO2 adsorbed by chemisorption and amount of CO2 adsorbed by physisorption at 25°C calculated from the fitting parameters of the Dualsite Langmuir model. [file Table_1.docx]

**Table S1.** Relation between amount of CO_2_ adsorbed by chemisorption and amount of CO_2_ adsorbed by physisorption at 25° C calculated from the fitting parameters of the Dualsite Langmuir model

|  | **CO_2 chemisorbed_/CO_2 physisorbed_** | | |
| --- | --- | --- | --- |
| **P (kPa)** | **MCF-80-20A** | **MCF-100-50P** | **MCF-120-50T** |
| 1 | 27.54 | 394.37 | 361.26 |
| 5 | 6.62 | 106.05 | 73.89 |
| 10 | 3.74 | 56.29 | 37.81 |
| 15 | 2.77 | 38.76 | 25.77 |
| 20 | 2.28 | 29.80 | 19.75 |
| 25 | 1.98 | 24.37 | 16.14 |
| 30 | 1.78 | 20.72 | 13.73 |
| 40 | 1.54 | 16.12 | 10.72 |
| 50 | 1.39 | 13.35 | 8.92 |
| 60 | 1.29 | 11.50 | 7.71 |
| 80 | 1.17 | 9.17 | 6.21 |
| 100 | 1.09 | 7.77 | 5.30 |
